# A temperature-sensitive metabolic valve and a transcriptional feedback loop drive rapid homeoviscous adaptation in *Escherichia coli*

**DOI:** 10.1038/s41467-024-53677-5

**Published:** 2024-10-30

**Authors:** Loles Hoogerland, Stefan Pieter Hendrik van den Berg, Yixing Suo, Yuta W. Moriuchi, Adja Zoumaro-Djayoon, Esther Geurken, Flora Yang, Frank Bruggeman, Michael D. Burkart, Gregory Bokinsky

**Affiliations:** 1 Department of Bionanoscience, Kavli Institute of Nanoscience, Delft University of Technology, Delft, The Netherlands; 2 Department of Chemistry and Biochemistry, University of California, San Diego, CA USA; 3 Systems Biology Lab, AIMMS/ALIFE, Vrije Universiteit Amsterdam, Amsterdam, The Netherlands; 4 Present Address: Molecular Microbial Physiology Group, Swammerdam Institute for Life Sciences, Faculty of Science, University of Amsterdam, Amsterdam, The Netherlands; 5 Present Address: Department of Immunopathology, Sanquin Research Amsterdam, Amsterdam, The Netherlands

**Keywords:** Regulatory networks, Metabolic pathways, Membranes, Bacterial physiology

## Abstract

All free-living microorganisms homeostatically maintain the fluidity of their membranes by adapting lipid composition to environmental temperatures. Here, we quantify enzymes and metabolic intermediates of the *Escherichia coli* fatty acid and phospholipid synthesis pathways, to describe how this organism measures temperature and restores optimal membrane fluidity within a single generation after a temperature shock. A first element of this regulatory system is a temperature-sensitive metabolic valve that allocates flux between the saturated and unsaturated fatty acid synthesis pathways via the branchpoint enzymes FabI and FabB. A second element is a transcription-based negative feedback loop that counteracts the temperature-sensitive valve. The combination of these elements accelerates membrane adaptation by causing a transient overshoot in the synthesis of saturated or unsaturated fatty acids following temperature shocks. This strategy is comparable to increasing the temperature of a water bath by adding water that is excessively hot rather than adding water at the desired temperature. These properties are captured in a mathematical model, which we use to show how hard-wired parameters calibrate the system to generate membrane compositions that maintain constant fluidity across temperatures. We hypothesize that core features of the *E. coli* system will prove to be ubiquitous features of homeoviscous adaptation systems.

## Abstract (web summary)

Free-living microorganisms regulate the fluidity of their membranes by adapting lipid composition to environmental temperatures. Here, the authors quantify lipid biosynthetic enzymes and metabolic intermediates to provide a quantitative description of how the bacterium *Escherichia coli* maintains constant membrane fluidity after a temperature shock.

## Introduction

**Fig. 1:**
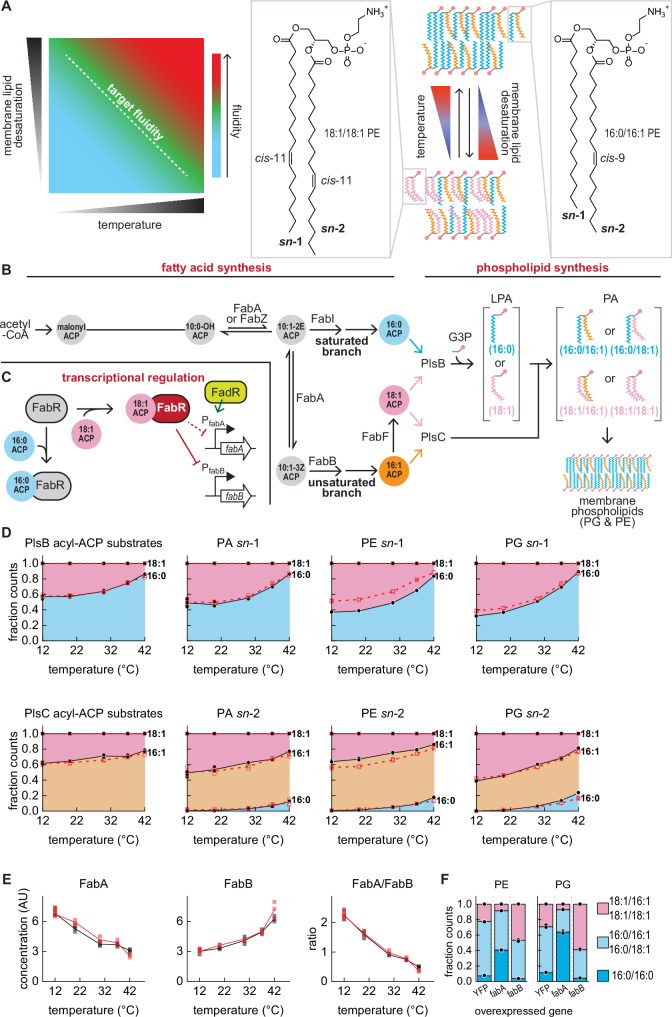
Homeoviscous adaptation and membrane metabolism in *E. coli.* **A** *E. coli* maintains membrane fluidity by titrating the fraction of membrane phospholipids bearing unsaturated fatty acids. **B** The *E. coli* fatty acid synthesis pathway splits into saturated and unsaturated branches at the enoyl-acyl-ACP intermediate C10:1(2*E*) ACP. Reduction by the enoyl-acyl-ACP reductase FabI initiates synthesis of saturated fatty acid thioesters (primarily C16:0 ACP). Alternatively, reversible isomerization of C10:1(2*E*) ACP by the bifunctional hydroxyl-acyl-ACP dehydratase/enoyl-acyl-ACP isomerase FabA generates C10:1(3*Z*) ACP, a substrate for the β-keto-acyl-ACP synthase FabB. As C10:1(2*E*) and C10:1(3*Z*) ACP are reversibly interconverted by FabA, FabI and FabB indirectly compete for a common pool of substrates. The FabB reaction initiates synthesis of the unsaturated fatty acid thioester C16:1 ACP. A fraction of the C16:1 ACP pool is elongated by the β-keto-acyl synthase enzyme FabF, ultimately generating C18:1 ACP. Phospholipids are synthesised by PlsB and PlsC, which transfer acyl groups to glycerol-3-phosphate (G3P) to produce phosphatidic acid (PA). The PA headgroup is further modified to yield membrane phospholipids phosphatidylglycerol (PG) and phosphatidylethanolamine (PE). Complete pathways are depicted in Supplemental Fig. 1. **C** C16:0 and C18:1 ACP compete for binding to the transcriptional regulator FabR. The FabR-C18:1 ACP complex represses *fabB* transcription, while FabR binding to C16:0 ACP relieves repression. FabA expression is primarily controlled in response to fatty acyl-CoA generated from exogenous fatty acids by the transcriptional regulator FadR^33^. **D** LCMS quantification of long-chain acyl-ACP, PA, PG, and PE. Symbols are LCMS counts measured from one technical replicate (of 3 total) from 2 independent cultures (cultures distinguished by solid black and open red symbols). Lines indicate the average obtained for the cultures (solid black and dashed red lines). Values are normalised to fraction of total LCMS counts per unit biomass (determined by optical density (OD)), which approximates relative concentrations. Areas represent total LCMS counts for phospholipid species bearing acyl chains at either the *sn-*1 or *sn-*2 positions. **E** Relative abundance of FabA and FabB enzymes in cultures maintained at 5 temperatures. 3 technical measurements (symbols) and their average (line) are depicted for 2 independent cultures (distinguished by colour) at each temperature. **F** Phospholipid compositions of strains overexpressing YFP (control), FabA, and FabB enzymes. Bars depict average of 3 technical measurements (depicted by symbols) from one culture each. Source data are provided as a Source Data file.

Lipid membranes provide living cells with a semi-permeable barrier, a platform for cell wall assembly, and a channel for electron transport. These and many other essential functions are strongly affected by the membrane fluidity (or viscosity), a physical property that is highly sensitive to temperature^1^. Specifically, low temperatures reduce membrane fluidity by increasing the packing of membrane lipids. Organisms counteract the effects of temperature by varying the proportion of lipids that disrupt membrane packing such as unsaturated or branched-chain fatty acids (Fig. 1A) or by varying fatty acyl chain length. This response, known as homeoviscous adaptation, maintains cell membranes at a fixed viscosity level across all growth temperatures^2^. A variety of membrane sensors, metabolic pathways, and transcriptional regulators required for homeoviscous adaptation have been described in prokaryotic and eukaryotic microorganisms^3–5^ and multicellular organisms^6^. How these components stabilise membrane fluidity is poorly understood.

In *Bacillus subtilis* and model eukaryotes, membrane composition control is directly linked with membrane fluidity. Membrane properties are monitored by integral protein sensors that interact with transcriptional pathways^7,8^. These pathways control expression of enzymes that change membrane composition by modifying existing membrane lipids (*B. subtilis*^9^) or lipid precursors (*Saccharomyces cerevisiae*^10^). In contrast, membrane fluidity and composition control are indirectly connected in *Escherichia coli. E. coli* does not monitor membrane fluidity and controls its membrane composition by varying the composition of the pool of fatty acid precursors from which phospholipids are synthesised (Fig. 1B)^11^. *E. coli* saturated and unsaturated fatty acids are produced as thioesters attached to an acyl carrier protein (acyl-ACP) (Fig. 1B, Supplemental Fig. 1). The first enzyme of the phospholipid synthesis pathway (PlsB) transfers a C16:0 or C18:1 fatty acid to the *sn-*1 position of glycerol-3-phosphate (G3P), followed by PlsC-catalysed acyl transfer from C16:1 or C18:1 ACP to *sn-*2 to generate phosphatidic acid (PA). Because PlsC chiefly transfers unsaturated fatty acyl chains whereas PlsB transfers both saturated and unsaturated fatty acyl chains, membrane fluidity is primarily determined by the fatty acid incorporated at *sn-*1 by PlsB. The composition of the fatty acid pool is also transcriptionally regulated by FabR and FadR, which control expression of branchpoint enzymes FabA and FabB (Fig. 1C)^12,13^. However, temperature also directly controls the fatty acid pool composition via a transcription-independent mechanism^14^. This effect is attributed to the activity of the β-keto acyl-ACP synthase FabF, which initiates synthesis of C18:1 ACP from C16:1 ACP^15,16^. How transcriptional regulation and the temperature sensitivity of fatty acid synthesis each contribute to homeoviscous adaptation is unclear.

Here, we use a systems approach to determine how *E. coli* achieves homeoviscous adaptation. By comprehensively quantifying enzymes and metabolites of the fatty acid and phospholipid synthesis pathways, we reveal how *E. coli* integrates a temperature-sensitive metabolic valve with a transcriptional feedback loop to accelerate response times and adapt membrane composition within a single cell cycle. We use a mathematical model to determine how the system is calibrated to produce specific membrane compositions for each temperature. We predict that homeoviscous adaptation systems in diverse organisms will feature similar regulatory motifs that accelerate adaptation to temperature shocks.

## Results

### Fatty acid synthesis enzyme concentrations exhibit paradoxical correlations with membrane lipid saturation

We prepared cultures of *E. coli* NCM3722 using defined minimal medium (MOPS/0.2% glycerol) at 5 temperatures from 12 to 42 °C. Acyl-ACP, proteins, and phospholipids were extracted from exponential-phase cultures and quantified using liquid chromatography/mass spectrometry (LCMS)^17,18^. Remarkably, the proportions of 16:0 and 18:1 *sn*-1 phospholipids closely correspond to the composition of the PlsB substrate pool: C16:0 ACP and 16:0 *sn*-1 phospholipids increase with temperature, while C18:1 ACP and 18:1 *sn*-1 phospholipids decrease (Fig. 1D and Supplemental Fig. 2A, B). Likewise, the proportions of 16:1 and 18:1 *sn*-2 phospholipids closely match the PlsC acyl-ACP substrate pool, with rare 16:0 *sn-*2 phospholipids increasing with temperature^19^. These measurements confirm that phospholipid composition is determined by the PlsB and PlsC substrate pools.

To determine how the substrate pools are controlled by transcriptional regulation, we quantified fatty acid pathway enzymes at each temperature. While concentrations of most enzymes remain relatively constant (Supplemental Fig. 2C), FabA decreases ~2-fold from 12 to 42 °C, while FabB increases ~2-fold (Fig. 1E). These trends contradict expectations as multiple studies report that increasing FabB or decreasing the FabA/FabB ratio at constant temperature increases unsaturated fatty acid production at the expense of saturated fatty acids^20–22^. We confirmed that FabA overexpression increases saturated acyl chains, while FabB overexpression increases unsaturated acyl chains (Fig. 1F).

### Acyl-ACP pool composition responds immediately after temperature shocks and overshoots final steady-state composition

**Fig. 2:**
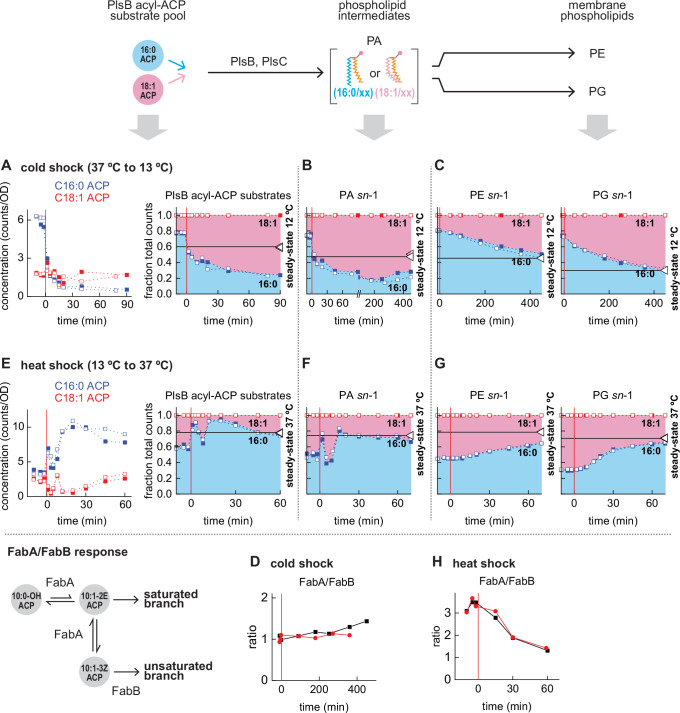
The fatty acid synthesis pathway responds immediately to changing temperature and exhibits overshoot kinetics. **A**–**C**, **E**–**G**. Pool compositions of PlsB acyl-ACP substrates (**A**, **E**), phospholipid intermediate PA (**B**, **F**), and membrane phospholipids PE & PG (**C**, **G**) during temperature shocks (0 min). Final steady-state values indicated by horizontal lines to highlight overshoot. **D**, **H** FabA/FabB ratios following cold shocks (**D**) and heat shocks (**H**). All kinetic series obtained from 2 independent experiments distinguished by closed and open symbols (**A**–**C**, **E**–**G**) or by colour (**D**, **H**) with each symbol depicting a single measurement. Source data are provided as a Source Data file.

The unexpected relationship between membrane composition and the FabA/FabB ratio suggests that PlsB and PlsC substrate pools are influenced by non-transcriptional mechanisms. Cold temperatures are known to increase unsaturated fatty acid production via a rapid post-translational response^14^. To resolve the rapid response, we subjected cultures to a rapid cold shock from 37 to 13 °C (Supplemental Fig. 3A). The cold shock immediately altered both acyl-ACP and phospholipid intermediate pools and arrested growth for 1 h (Supplemental Fig. 3B&C). Strikingly, within 5 min of the cold shock C16:0 ACP decreased approximately 5-fold, while C18:1 ACP remained stable (Fig. 2A). As a result, C18:1 ACP becomes the most abundant PlsB substrate. The phospholipid synthesis pathway immediately reflects this change: 18:1 *sn*-1 PA increased at the expense of 16:0 *sn-*1 PA (Fig. 2B and Supplemental Fig. 4A). Intriguingly, the C18:1 ACP fraction immediately after the cold shock far exceeds its size during steady-state growth at 12 °C (horizontal lines in Fig. 2A), an overshoot reflected in the PA pool that persists for at least 7 h (Fig. 2B). Remarkably, the 18:1 *sn*-1 fractions of PG and PE reach final steady-state 12 °C levels within 8 h, approximately one doubling period in these conditions (7 h) (Fig. 2C).

The rapid response of the acyl-ACP pools is characteristic of post-translational regulation^14^. We quantified fatty acid synthesis enzymes to determine whether transcriptional regulation also contributes to the cold shock response. Up to 7.5 h after the cold shock, concentrations of all enzymes remain stable aside from FabB, which after 5 h begins to decrease and increase the FabA/FabB ratio (Fig. 2D, Supplemental Fig. 4B). As the FabA/FabB ratio does not substantially change until long after the cold shock, the adaptations observed are achieved by post-translational regulation.

We next subjected cultures to a heat shock (13 °C to 37 °C). Cell growth immediately accelerated after the heat shock (Supplemental Fig. 3D). The fatty acid and phospholipid intermediate pools responded within 1 min, with most acyl-ACP following trajectories that completely reverse cold shock behaviours (Fig. 2E, F and Supplemental Figs. 3C and 4A). Notably, the heat shock increases C16:0 ACP more than 2-fold while C18:1 ACP decreases by 2-fold; as a result, C16:0 ACP becomes the most abundant PlsB substrate within 2 min, which in turn increases C16:0 *sn-*1 PA (Fig. 2E, F). Similarly to the C18:1 ACP fraction following cold shocks, the C16:0 ACP fraction of the PlsB substrate pool briefly overshoots its steady-state value at 37 °C. C16:0 *sn-*1 PG closely approaches steady-state levels within one doubling period (58 min), while the membrane phospholipid PE approaches steady-state more slowly (Fig. 2G and Supplemental Fig. 4A).

Following the heat shock, the concentrations of all enzymes monitored remained stable aside from FabA and FabB (Supplemental Fig. 4C). FabA and FabB did not substantially change within 15 min of the heat shock, indicating that the initial response is driven by a post-translational mechanism. FabB increased after 15 min and reached ~2-fold of the pre-shock level after 60 min, whereas FabA decreased slightly. As a consequence, the FabA/FabB ratio decreased ~2-fold, nearly reaching the steady-state ratio observed at 37 °C (Fig. 2H). Given that higher FabB expression increases unsaturated fatty acid synthesis, the FabB response is likely driving the increased unsaturated fatty acid synthesis observed after the first 15 min. The delayed FabB response thus corrects the excess production of saturated fatty acids occurring immediately following the heat shock. Therefore, the apparent paradox of FabA and FabB concentration trends observed during steady-state growth can be understood as a transcriptional response that partially counteracts the post-translational response to temperature.

### Temperature shifts reroute fatty acid flux between saturated and unsaturated branches by tuning relative fluxes through FabB and FabI

**Fig. 3:**
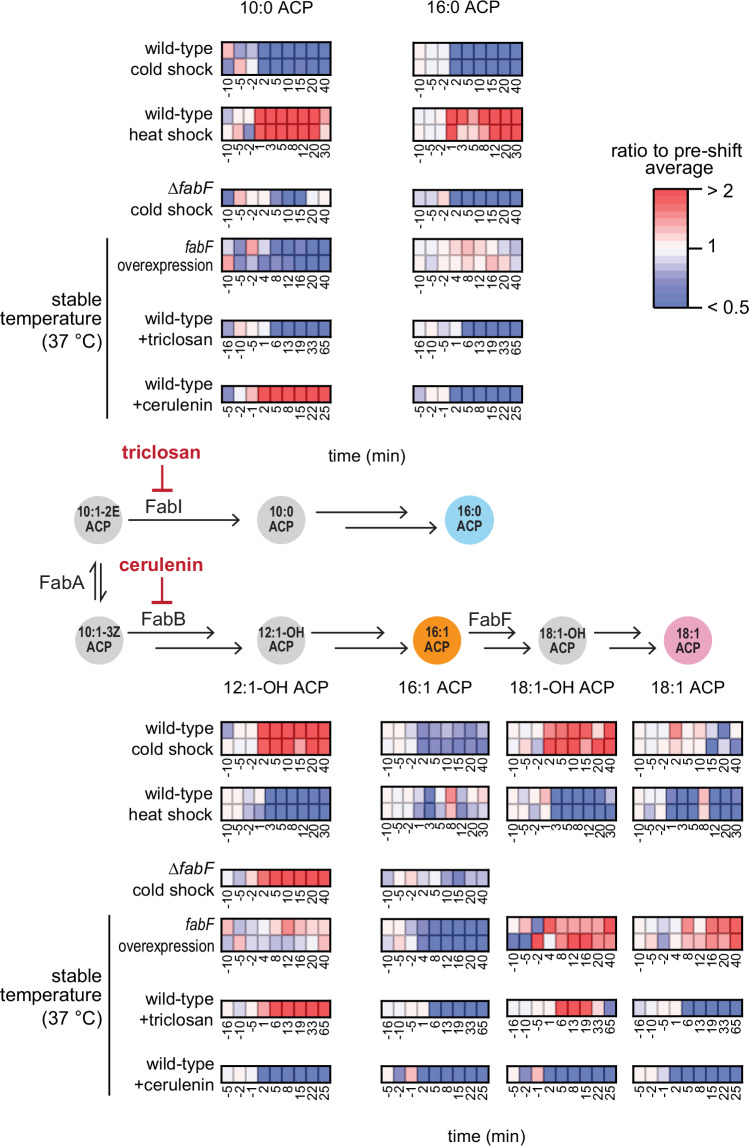
Temperature control of fatty acid synthesis. Comparison of acyl-ACP dynamics following temperature shocks, cold shock in a Δ*fabF* strain, *fabF* overexpression in wild-type *E. coli*, FabI inhibition (triclosan), and FabB inhibition (cerulenin). Values for each time point are normalised by the average value obtained from the 3 measurements preceding the perturbation. For temperature shocks with the wild-type strain and the *fabF* overexpression experiment, two independent cultures are depicted. For triclosan and cerulenin, data depicted are representative of two independent cultures. Data from the Δ*fabF* cold shock are obtained from one culture. Source data are provided as a Source Data file.

Temperature-induced adaptations in *E. coli* are proposed to require FabF, which initiates synthesis of the PlsB substrate C18:1 ACP^15,23^. Because C16:1 thioesters are poor substrates for PlsB relative to C18:1 thioesters, *E. coli* Δ*fabF* should be less able to generate unsaturated *sn*-1 phospholipids^24^. FabF-catalysed elongation of C16:1 ACP is relatively temperature-insensitive, a property thought to increase C18:1 ACP in cold temperatures^15,16^. However, our data indicate that cold shocks adapt membrane phospholipids not by increasing C18:1 ACP, but rather by decreasing C16:0 ACP (Fig. 2A). Furthermore, temperature shocks affect acyl-ACP pools throughout the fatty acid synthesis pathway beyond the C16:1 ACP elongation step (Supplemental Figs. 3C and 5A). To test whether increasing FabF activity depletes C16:0 ACP in a manner resembling a cold shock, we placed *fabF* under control of an inducible promoter and monitored acyl-ACP dynamics following *fabF* induction. While *fabF* overexpression increased C18:1 ACP relative to C16:1 ACP, the proportion of C18:1 ACP within the PlsB substrate pool did not substantially increase; nor did C16:0 ACP decrease (Fig. 3 and Supplemental Fig. 5B).

To further examine whether FabF contributes to temperature adaptation, we subjected *E. coli* Δ*fabF* to a cold shock. As observed in the wild-type strain, C16:0 ACP decreased by 4-fold (Fig. 3). In fact, most acyl-ACP in *E. coli* Δ*fabF* responded to the cold shock similarly to wild-type: acyl-ACP products of FabI decrease, while hydroxyl-acyl-ACP in the unsaturated branch increase (Supplemental Fig. 5A). Next, we analysed the steady-state phospholipid composition of *E. coli* Δ*fabF* at 12, 20, and 37 °C. While 18:1 *sn-*1 phospholipids are nearly absent, 16:1/16:1 membrane phospholipids, which are rare in the wild-type strain (<3% total PG), become abundant. This is likely caused by the high proportion of C16:1 ACP in the *E. coli* Δ*fabF* strain overcoming substrate preferences of PlsB (Supplemental Fig. 5C). Interestingly, the proportion of 16:1 *sn*-1 in *E. coli* Δ*fabF* increases at cold temperatures, while conversely 16:0 *sn-*1 phospholipids increase at 37  °C relative to cold temperatures (Supplemental Fig. 5C). Our data agree with previous measurements of *E. coli* Δ*fabF* phospholipid composition^25^, which also indicate that 16:0 acyl chains increase with temperature (Supplemental Fig. 5D). Importantly, our results reveal that *E. coli* Δ*fabF* retains the post-translational temperature response and a membrane composition that changes across temperatures. These are consistent with fatty acid synthesis reconstitution experiments using purified enzymes, which found that FabF titration did not change the ratio between saturated and unsaturated fatty acid production^26^.

We considered an alternative mechanism in which temperatures adapt acyl-ACP pools by reallocating fluxes into the saturated and unsaturated pathways, which indirectly compete for a pool of substrates (C10:1(2*E*) and C10:1(3*Z*) ACP) undergoing reversible isomerization by FabA. In this mechanism, cold temperatures restrict saturated fatty acid synthesis by decreasing the rate of C10:1(2*E*) ACP reduction by FabI relative to C10:1(3*Z*) ACP elongation by FabB. To test this alternative, we monitored acyl-ACP pools immediately after inhibiting FabI with triclosan^27^. Similarly to the cold shock, triclosan decreased C16:0 ACP and C16:1 ACP and increased C18:1-OH ACP (Fig. 3). Interestingly, most acyl-ACP responded to triclosan in a manner similar to cold shock: FabI products consistently decreased, while hydroxyl-acyl-ACP within the unsaturated pathway increased and >10-carbon saturated hydroxyl-acyl-ACP decreased (Supplemental Fig. 5A). Furthermore, both triclosan and cold shock decrease the FabI product C10:0 ACP while increasing an early unsaturated pathway intermediate (C12:1-OH ACP) (Fig. 3). We next compared FabB inhibition to heat shock. Both heat shock and the FabB inhibitor cerulenin similarly affect C10:0 ACP and C12:1-OH ACP and caused accumulation of FabB and FabF substrates within the saturated branch (Fig. 3 and Supplemental Fig. 5A). These similarities suggest that temperature allocates flux between saturated and unsaturated fatty acid branches by varying flux through FabI and FabB.

How does temperature vary flux allocation between saturated and unsaturated branches? A simple explanation consistent with our data would be that FabI and FabB activities exhibit different temperature dependencies. Initial in vitro comparisons of FabI and FabB activities at 27 and 37 °C using C10:1(2*E*) and C10:1(3*Z*) ACP substrates indicates that FabI exhibits approximately 2-fold less activity at 27 °C; however FabB activity assays are not conclusive (Supplemental Fig. 5E). Mathematical models of branch point metabolism suggest several alternative mechanisms involving temperature-dependent activities of FabA and FabZ that also enable temperature-dependent flux reallocation between saturated and unsaturated pathways (Supplemental Note 1).

### A simple mathematical model recapitulates temperature dependence of steady-state membrane composition

**Fig. 4:**
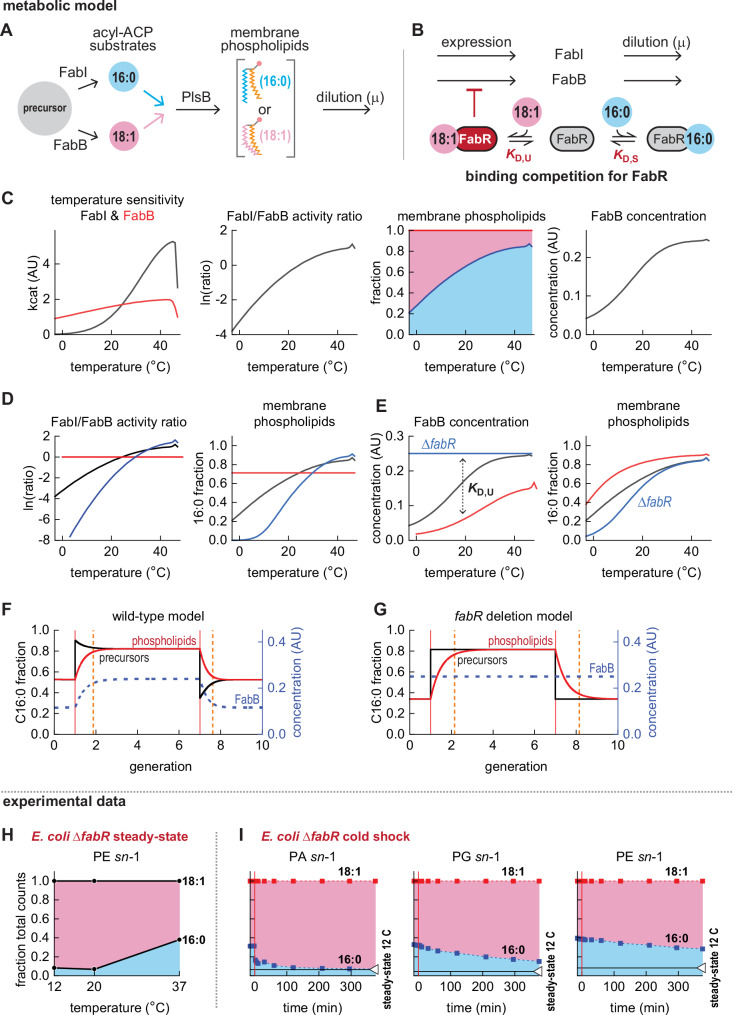
A mathematical model recapitulates core behaviours of the *E. coli* homeoviscous adaptation system. **A** Simulated phospholipid synthesis from saturated (16:0) and unsaturated acyl-ACP (18:1). **B** Simulated regulation of FabB expression by C18:1 ACP-FabR, with C16:0 ACP competing for FabR and relieving *fabB* repression. **C** Asymmetric temperature sensitivities of FabB and FabI increases both saturated phospholipids and FabB concentration with temperature. **D** Varying FabI and FabB temperature sensitivities changes how the phospholipid composition responds to temperature. **E** Varying C18:1 ACP affinity for FabR changes the relationship between temperature and FabB expression, which in turn affects phospholipid composition. **F** Simulated responses to temperature shocks exhibit overshoot kinetics and accelerated adaptation of the phospholipid pool, achieving 90% adaptation within 1 generation (indicated by dashed orange line) **G** A Δ*fabR* model does not exhibit overshoot kinetics and reaches steady-state membrane composition more slowly than wild-type. **H** Steady-state membrane composition of *E. coli* Δ*fabR*. **I** Cold shock of a Δ*fabR* knockout confirms model predictions: without transcriptional repression by FabR, no overshoot kinetics are possible and homeoviscous adaptation requires more than one generation time. Steady-state data from three samples obtained from one culture prepared at each temperature. Kinetic series obtained from one experiment; symbols depict individual measurements. Source data are provided as a Source Data file.

Our experiments reveal two contributors to homeoviscous adaptation: (1) a post-translational mechanism that allocates flux between saturated and unsaturated pathways according to temperature, and (2) a transcriptional response that shifts FabB expression to partially counteract the post-translational mechanism. The delayed increase in FabB upregulation following heat shock (Fig. 2H) and decreased levels of FabB at cold temperatures (Fig. 1E) are both consistent with repression of *fabB* transcription responding to variation in concentrations of the C18:1 ACP-FabR repressor complex^12^. To test whether temperature sensitivity and FabR regulation are sufficient to reproduce our observations, we built a differential equation-based model that simulates a temperature-controlled branched pathway with a transcriptional feedback loop. The branched pathway consists of two enzymes (FabI and FabB) that produce saturated and unsaturated precursors from a common substrate. Both precursors are converted to membrane phospholipids at rates according to their relative abundance (Fig. 4A). We placed FabB expression under control of a modelled FabR, which represses FabB when bound to the unsaturated precursor. The saturated and unsaturated precursors each compete for FabR (Fig. 4B). Further details are provided in Supplemental Note 2.

We introduced an asymmetric temperature dependency in the modelled FabI and FabB product formation rates such that FabI generates less product in cold temperatures compared to warm temperatures, while the rate of product formation by FabB remains relatively stable across temperatures. This causes unsaturated membrane phospholipids to decrease with increasing temperature (Fig. 4C). Decreased concentrations of unsaturated precursors relieves FabB repression by FabR, which in turn increases FabB. Thus, our model captures the essential observed steady-state behaviours of homeoviscous adaptation. Next, we tuned our model to explore how the temperature dependency of membrane composition might be calibrated by evolution. Varying the temperature sensitivity of FabI changes the temperature sensitivity of the FabI/FabB activity ratio, which in turn adjusts how phospholipid composition varies with temperature (Fig. 4D). The membrane composition becomes temperature-insensitive when FabI and FabB product formation rates change identically with temperature. Therefore, an asymmetric temperature dependence in branch point fluxes is both necessary and sufficient to cause membrane composition to vary with temperature.

We next sought to determine how transcriptional regulation by FabR contributes to steady-state membrane composition. Tuning the affinity of the FabR regulator for the unsaturated acyl-ACP precursor shifted the temperature sensitivity of FabB abundance and the saturated phospholipid fraction (Fig. 4E). Eliminating the interaction between FabR and the unsaturated precursor (equivalent to a Δ*fabR* strain) abolishes the temperature sensitivity of FabB expression, which remains at maximum concentration. This “Δ*fabR* model” retains temperature control of membrane composition but increases the fraction of unsaturated phospholipids (Fig. 4E). Therefore, both transcriptional regulation parameters and the temperature sensitivities of FabI and FabB may be calibrated to produce specific membrane compositions across temperatures.

### Transcriptional regulation by FabR accelerates homeoviscous adaptation

Models lacking FabR regulation retain temperature control of membrane composition. This raises questions about the utility of FabR: why retain transcriptional regulation when a temperature-sensitive branch point is sufficient to adapt membrane composition? A role for transcriptional regulation is suggested by the delayed response of FabB levels following temperature shocks. The immediate response driven by temperature control of the FabI/FabB flux ratio initiates adaptations by producing unsaturated (or saturated) fatty acids at levels that exceed final steady-state proportions. Over time, transcriptional regulation partially counteracts the overshoot by tuning FabB expression, thus steering the acyl-ACP pool to its steady-state composition. However, the initial excess production of saturated or unsaturated fatty acids accelerates adaptation. FabR may be retained to enable overshoot kinetics that accelerate membrane adaptation.

We first tested whether a “wild-type” model (featuring both temperature regulation of FabI/FabB flux and FabB repression by FabR) could exhibit overshoot kinetics. In response to a heat shock, the saturated precursor fraction initially surpassed the final steady-state value, mimicking the experimentally observed overshoot (Fig. 4F). The overshoot decreased repression of FabB expression by FabR, causing FabB concentration to increase, which in turn increased unsaturated precursor synthesis. This eliminated the overshoot and steered the composition of the precursor pool to its final value. A simulated cold shock triggered an overshoot response in the opposite direction. For both simulated temperature shocks, the membrane phospholipid pool reached within 90% of its final composition within 1 generation following the heat shock.

We next simulated the response of a pathway retaining differential temperature regulation of FabI/FabB activity but lacking transcriptional regulation by FabR (Δ*fabR* model). Subjecting the Δ*fabR* model to a simulated heat shock immediately shifted the precursor pool to its final steady-state composition (Fig. 4G). No overshoot was observed as FabB expression is insensitive to the precursor pool. A simulated cold shock also exhibited a stepwise change in precursor abundance. Despite retaining the immediate response of the temperature-sensitive branch point, the Δ*fabR* model required more time than the wild-type strain to reach 90% of its final steady-state composition at the new temperature.

We tested our model by characterising the steady-state membrane composition of *E. coli* Δ*fabR* at 3 temperatures. As previously observed^28^ and as reflected in our model, unsaturated membrane phospholipids increased in *E. coli* Δ*fabR* relative to wild-type while retaining temperature-dependent composition (Fig. 4H and Supplemental Fig. 6A). Consistent with our model, FabB expression increased approximately 4-fold in *E. coli* Δ*fabR*, which decreases the FabA/FabB ratio relative to the wild-type ratio (Supplemental Fig. 6B). Increased 18:1 phospholipids in *E. coli* Δ*fabR* require FabF and are not due to elongation of C16:1 ACP by FabB (Supplemental Fig. 6C). To test whether FabR is necessary for overshoot kinetics as predicted by our model, we subjected *E. coli* Δ*fabR* to a cold shock. Consistent with our model, the cold shock immediately increased the proportion of 18:1 *sn*-1 PA intermediates without an overshoot and the proportions of 18:1 *sn*-1 PG and PE did not reach final steady-state values within one generation time (7.5 h) (Fig. 4I and Supplemental Fig. 6D). This confirms that FabR enables overshoot kinetics following temperature shocks and accelerates membrane adaptation.

### Rapid homeoviscous adaptation accelerates growth recovery following cold shocks in the respiration-dependent medium

The evolution of FabR implies that the ability to accelerate homeoviscous adaptation is advantageous. However, the benefits of homeoviscous adaptation have proven elusive in experimental settings. While extreme perturbations of membrane composition reduce viability, smaller perturbations exert no obvious effect^29–31^. For example, due to its inability to synthesise *cis-*vaccenate, *E. coli* Δ*fabF* is less able to vary membrane fluidity but grows at low temperatures^32^ and recovers quickly from cold shocks^24^. These findings raise the question of how rapid homeoviscous adaptation provides an evolutionary advantage.

**Fig. 5:**
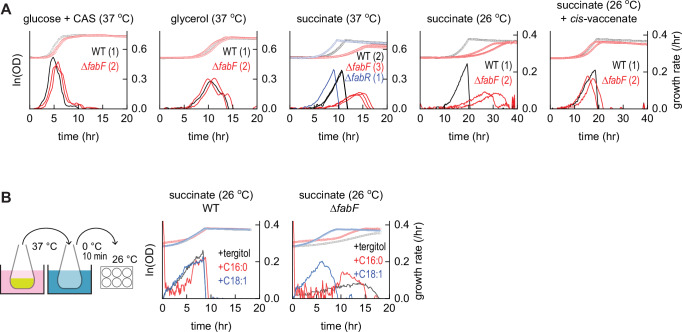
Increased synthesis of 18:1 phospholipids accelerates growth recovery from cold shock in respiration-dependent conditions. Growth monitored in a 6-well plate by optical density (symbols). Derivatives of optical density measurements (smoothed over 7 points) yields instantaneous growth rates (lines); each line represents an independent culture. **A** Comparison of wild-type (WT), Δ*fabR*, and Δ*fabF* growth in defined media containing indicated carbon sources at indicated temperatures (0.2% w/vol glucose + 0.1% w/vol CAS amino acids, 0.2% glycerol, or 0.2% succinate). The number of independent cultures is indicated for each experiment. **B** Comparison of wild-type and Δ*fabF* (one experiment for each condition) following cold shock from 37 °C to 0 °C and subsequent recovery at 26 °C in a microplate reader with tergitol carrier (control), palmitate (16:0 fatty acid), or *cis-*vaccenate (18:1 fatty acid). Source data are provided as a Source Data file.

A recent study revealed that the electron transport reactions of respiration are highly sensitive to membrane fluidity^21^. Succinate catabolism (oxidation to fumarate) requires membrane diffusion of a ubiquinone. To test whether homeoviscous adaptation is required for optimal steady-state growth in respiration-dependent conditions, we compared growth of *E. coli* Δ*fabF* against wild-type in several defined media. Consistent with prior observations^24,32^, *E. coli* Δ*fabF* grew normally in both glucose and glycerol medium, but exhibited severely reduced growth in succinate medium (Fig. 5A). Robust growth of *E. coli* Δ*fabR* in succinate medium likely reflects its high membrane fluidity as unsaturated lipids in *E. coli* Δ*fabR* exceed wild-type proportions (Fig. 4H). To confirm that the growth defect of *E. coli* Δ*fabF* is caused by decreased membrane fluidity, we added the fatty acid *cis-*vaccenate, which is converted by FadD to the PlsB substrate C18:1-CoA. *Cis-*vaccenate completely restored growth of the Δ*fabF* strain to match the wild-type strain (Fig. 5A). This suggests that 18:1 *sn-*1 phospholipids may be more effective at maintaining membrane fluidity than the 16:1 *sn-*1 phospholipids generated by *E. coli* Δ*fabF* at cold temperatures.

We next tested whether restoring membrane fluidity with *cis-*vaccenate accelerates cold shock recovery. Wild-type and Δ*fabF* succinate cultures prepared at 37 °C were transferred to an ice bath and incubated for 10 min before culturing at 26 °C. As expected, *E. coli* Δ*fabF* grew extremely slowly following the cold shock unless supplemented with *cis-*vaccenate, which enabled immediate growth recovery and a steady-state growth rate that matched wild-type (Fig. 5B). The saturated fatty acid palmitate did not recover growth, indicating that 18:1 phospholipid synthesis is required to restore growth.

## Discussion

**Fig. 6:**
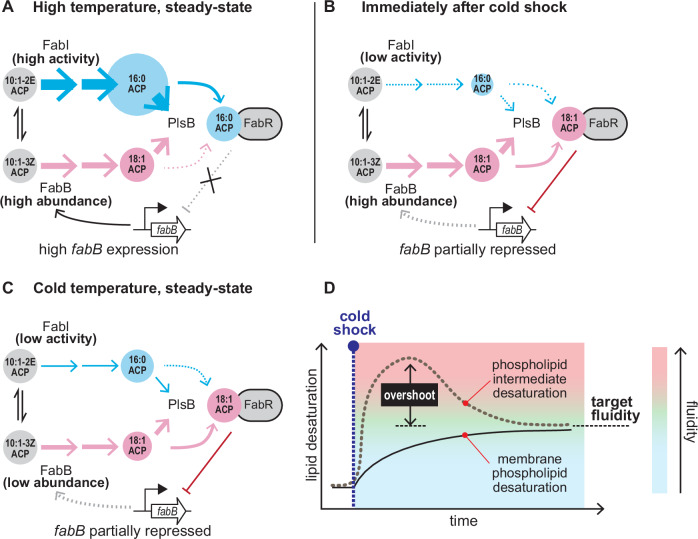
An incoherent feed-forward loop accelerates membrane adaptation to temperature shocks. **A** In warm temperatures, the balance between FabI and FabB fluxes favours FabI, leading to a higher proportion of saturated fatty acids. The high abundance of C16:0 ACP relative to C18:1 ACP reduces repression of *fabB* by favouring the non-repressing FabR-C16:0 ACP complex. **B** Cold shocks immediately reduce flux entering the saturated branch via FabI. This reduces production of saturated fatty acids relative to unsaturated fatty acids, leading to a rapid increase in unsaturated phospholipid production from the adapted fatty acyl-ACP pool to levels that exceed the eventual steady-state levels. The overshoot is corrected via transcriptional regulation: decreased C16:0 ACP favours formation of FabR-C18:1 ACP, which represses *fabB*. Subsequently, FabB concentration is reduced by dilution through growth, thus decreasing synthesis of unsaturated fatty acids until the final steady-state balance of saturated and unsaturated fatty acids is achieved (**C**). These dynamics result in overshoot kinetics (**D**) that accelerate membrane adaptation.

The broad conservation of homeoviscous adaptation indicates that the ability to adjust membrane composition in response to temperature is an essential trait in natural environments. How organisms calibrate membrane composition is poorly understood. Several aspects of the homeoviscous adaptation system in *E. coli* have been identified, including the control of membrane composition by the acyl-ACP pool^11^ and direct temperature control of unsaturated fatty acid synthesis via a transcription-independent mechanism^14^, while the role of transcriptional regulation by FabR has remained obscure^12,28^. Our study reveals how these components are integrated into a system that measures temperature, adapts fatty acid synthesis, and rapidly restores membrane fluidity (Fig. 6). These insights are made possible by combining comprehensive measurements of metabolites and enzymes of the fatty acid and phospholipid pathways with a quantitative model that reproduces the core properties of homeoviscous adaptation in *E. coli*.

Our data significantly revise the previous model of *E. coli* homeoviscous adaptation. We find that the enzyme FabF is not required to initiate temperature-driven changes in the proportions of saturated and unsaturated membrane phospholipids. Instead, FabF augments temperature adaptation by synthesising 18:1 fatty acid, which appears to be more effective at adapting the membrane to cold temperatures than 16:1 fatty acid. We find rather that temperature directly determines the flux partitioned between the saturated and unsaturated fatty acid synthesis pathways. Our data suggest this is implemented by different temperature sensitivities of FabI and FabB. Consistent with this mechanism, our in vitro experiments indicate that FabI activity is reduced at lower temperatures. Demonstrating that the rate of FabB-catalysed elongation of C10:1(3*Z*) ACP remains stable despite changes in temperature would provide strong support for this mechanism. However, our in vitro experiments with FabB are inconclusive as we were unable to monitor the early kinetics of the elongation reaction, possibly due to our use of the nonphysiological substrate malonyl-CoA instead of malonyl-ACP. Alternatively, temperature-dependent flux partitioning may be accomplished by changing FabA or FabZ activity, which our simulations indicate would alter the abundance of C10:1(2*E*) and C10:1(3*Z*) ACP. However, flux partitioning is achieved using temperature as a control mechanism to link the supply of unsaturated fatty acids to the demand for membrane fluidity. Our model suggests the temperature-dependent activities and expression levels of FabB and FabI may be calibrated by evolution to produce specific membrane compositions that stabilise membrane viscosity at any growth temperature.

The implementation of temperature control within the fatty acid pathway rather than a hypothetical temperature-sensitive transcriptional regulator ensures an immediate response to temperature shocks. This acceleration is important because *E. coli* cannot adjust fluidity by directly modifying its membrane lipids. Therefore, restoring membrane fluidity requires substantially diluting ~10^7^ membrane phospholipids with phospholipids assembled from an adapted fatty acid pool. However, a temperature-sensitive metabolic pathway is insufficient on its own to adapt the membrane to new temperatures within a single generation. Counterintuitively, the accelerated adaptation we observe is made possible by a slow-responding feedback loop implemented by FabR^12,28,33^. Negative autoregulation of the unsaturated fatty acid pathway allows the initial temperature shock response to overshoot the eventual steady-state target. This overshoot is corrected by FabR, which adjusts FabB expression. Importantly, steady-state FabB concentrations are adjusted at all temperatures to ensure that cold and heat shocks always generate overshoot kinetics. The combination of two opposing regulatory components that respond at different timescales is a recurring biological motif that accelerates adaptation^34^.

We hypothesise that the general principles revealed here exist in any microorganism capable of rapid homeoviscous adaptation. Specifically, we predict that all homeoviscous adaptation systems will feature an acceleration mechanism. Responses similar to overshoot kinetics in membrane desaturation rates (referred to as “hyperinduction”) have been observed in prokaryotes with Des-type membrane fluidity sensors (*Bacillus megaterium and B. subtilis*)^35–37^, and in multicellular eukaryotes^6^, which use systems very different from *E. coli*. Membrane fluidity in the pathogen *Listeria monocytogenes* is modulated by tuning the branched structure of fatty acids^38^. The abundance of fluidity-enhancing fatty acids is at least partly controlled by the temperature-sensitive substrate preference of the *L. monocytogenes* FabH homologue^39^. This may provide a mechanism analogous to the FabI/FabB branch point in the *E. coli* pathway. Mathematical modelling of the *L. monocytogenes* fatty acid pathway based upon FabH substrate selectivity alone was unable to reproduce experimental observations, leading the authors of the work to suggest an additional step required to adapt membrane composition^40^. We suggest that *L. monocytogenes* homeoviscous adaptation may involve a slow-responding regulatory component that accelerates adaptation time (analogous to FabR) yet to be discovered.

## Methods

### Culture conditions

Unless otherwise indicated, cultures were grown in Erlenmeyer flasks with 0.2% glycerol / MOPS minimal medium with 0.2% (w/v) glycerol^41^. Flasks were incubated in a water bath (Grant Instruments Sub Aqua Pro) and stirred with a magnetic bar (1200 rpm) coupled to a magnetic plate (2mag MIXdrive 1 Eco and MIXcontrol 20). Optical density was measured using Ultrospec 10 Cell Density Meter (GE Healthcare). Fatty acid stock solutions (40 mM) were prepared by solubilisation in 26% tergitol and neutralisation to pH 7 using NaOH. For temperature shifts, 25-35 mL medium was transferred to an empty Erlenmeyer flask incubated in a water bath.

### Plasmids and strains

*Escherichia coli* K-12 strain NCM3722 (CGSC #12355) was used for all experiments. Δ*fabR* and Δ*fadF* strains were constructed using lambda-red recombination^42^. *fabF*, *fabB* and *fabA* were amplified from *E. coli* genomic DNA and cloned into BglBrick plasmid pBbA2k^43^ using restriction digestion and ligation. All primers used are listed in Supplemental Table 3.

### Culture sampling and LCMS analysis

Culture sampling and analyses of acyl-ACP, phospholipids, and proteins were performed largely as described in ref. ^18^. In brief, samples for all analysis methods were removed from cultures and rapidly quenched by adding directly to a 10% solution of trichloroacetic acid (2% final concentration). Quenched samples were pelleted by centrifugation and stored at -80 °C until analysis. For acyl-ACP and proteomics analysis, samples were lysed by suspending quenched pellets in a lysis solution with ^15^N-labelled internal standards before protein precipitation and digestion by Glu-C protease (acyl-ACP analysis) or trypsin (proteomics analysis). ^15^N-labelled internal standards were generated using U-^15^N *E. coli* whole cell extracts from MOPS minimal medium cultures with ^15^NH_4_Cl as the sole nitrogen source. For phospholipid analysis, phospholipids were extracted in MTBE solution^44,45^ pre-mixed with internal standards consisting of a phospholipid extract from a ^13^C-labelled culture grown in minimal MOPS medium with 0.2% U-^13^C glucose as the sole carbon source.

LC/MS quantification followed the methods described in ref. ^18^. For acyl-ACP analysis, frozen U-^15^N-labelled *E. coli* pellets were suspended in lysis buffer (10 mL of 50 mM potassium phosphate buffer, pH 7.2, 6 M urea, 10 mM N-ethyl-maleimide, 5 mM EDTA and 1 mM ascorbic acid) and added to TCA-quenched samples and ACP species were isolated by precipitation as described previously. Precipitated acyl-ACP were resuspended in 10 μL of digestion buffer (4% 2-octyl-glucoside in 25 mM potassium phosphate buffer, pH 7.2) and after adding 10 μL of 0.1 mg/mL GluC protease (Promega) incubated overnight at 37°C. After quenching by addition of 5 μL MeOH, samples were centrifuged and 10 μL was injected in LC/MS system. Separation was performed on 2.1 mm×50 mm 1.7 μm CSH C-18 column (Waters) held at 80°C using a binary gradient: 15% B, 3 min ramp to 25%, 9 min increase to 95% and 1 min hold at 95% B before 3 min re-equilibration at starting conditions (A: 25 mM formic acid, B: 50 mM formic acid) at a flow rate of 0.6 mL/min.

For phospholipid quantification, pelleted *E. coli* were resuspended in mixture containing 150 μL of MeOH, 250 μL of U-^13^C *E. coli* extract and 250 μL MTBE, and homogenised by vortexing and sonication. 125 μL of 15 mM citric acid/ 20 mM dipotassium phosphate buffer was added to homogenised pellets and vortexed. Liquid phases were separated by centrifugation for 10 min at 20000 g. 450 μL of the upper phase was moved to a new tube and dried in a vacuum centrifuge. Dried lipids were resuspended in 10 μL 65:30:5 (v/v/v) isopropanol/acetonitrile/H2O, supplemented with 10 mM acetylacetone. After addition of 5 μL H2O, 5 μL of resulting mixture was injected into the LC/MS system. Separation was performed on 2.1 mm×50 mm 1.7 μm CSH C-18 column (Waters) at 60°C with a flow rate of 0.6 mL/min using the following binary gradient: 25% B, ramp to 56%B in 6 min followed by linear increase to 80% B in 6 min, 2 min hold at 100% B and 3 min re-equilibration (A: 0.05% NH4OH in water, B: 0.05% NH4OH in 80% isopropanol 20% ACN).

For LC/MS targeted protein quantification, U-^15^N-labelled *E. coli* pellets suspended in 10 mL of 50 mM potassium phosphate buffer, pH 7.2 and 6 M urea were added to TCA-quenched experimental samples. Proteins were precipitated by chloroform/methanol precipitation and were resuspended in 10 μL of digestion buffer (4% 2-octyl-glucoside in 25 mM Tris buffer, pH 8.1 supplemented with 1 mM CaCl_2_ and 5 mM TCEP). Cysteine alkylation was achieved by adding 3 μL of 50 mM iodoacetamide followed by 15 min of incubation, after which 10 μL of 0.2 mg/mL Trypsin Gold (Promega) was added. Digestion proceeded overnight at 37 °C. 10 μL of digestion reaction was injected in LCMS system and separation performed on 2.1 mm × 50 mm 1.7 μm CSH C-18 column (Waters) held at 40°C using a binary gradient: 2% B, 20 min ramp to 25% B, 4 min increase to 40% B, 0.5 ramp to 80% and 1 min hold at 80% B before 3 min re-equilibration at starting conditions (A: 25 mL formic acid, B: 50 mM formic acid) at a flow rate of 0.5 mL/min.

LC/MS runs were performed using Agilent LCMS (binary pump (G1312B), autosampler (G7167A), temperature-controlled column compartment (G1316A), and triple quadrupole (QQQ) mass spectrometer (G6460C) equipped with a standard ESI source) all operated using MassHunter (version 7.0). Mass spectrometer set in dynamic MRM mode using transitions generated in silico by a script written in Python using RDkit library. Transitions for targeted proteomics assays were developed using Skyline^46^. LC/MS data was further processed in Skyline versions 4.x using an in silico generated transition list for targets and corresponding internal standards.

### Microplate experiments

All microplate experiments were performed with a Biotek Synergy HTX using 6-well plates. For experiments performed at stable temperatures, 2 mL of media was inoculated and plates continuously agitated (180 cm, 6 mm amplitude) with optical density (600 nm) recorded every 10 min. Fatty acids were added to 0.4 mM final concentration where indicated. For cold shock experiments, exponential-phase cultures were prepared in stir flasks maintained at 37 °C before transfer to Falcon tubes on ice. After 10 min of incubation, 2 mL were transferred to microplate wells and growth monitored using the microplate reader. For cold shock experiments, plates were continuously agitated as before with optical density recorded every 5 min.

### Activity measurements of FabI and FabB

Synthesis of *cis*-2-decenoate and acyl-ACP preparation are described in Supplemental Note 3. The FabB assay mixture contained 50 mM Tris (pH 7.4), 200 mM NaCl, 0.5 mM DTT, 2 mM malonyl-CoA, 40 μM 10:1(3-cis)-AcpP, and 20 μM FabB. Each reaction was incubated at specific temperatures for 5 min to equilibrate before initiation. 10 microliters of each reaction mixture were quenched by the addition of 3 μL of 6x urea loading dye at three different time points. Product formation was monitored by 15% urea-PAGE. The gel was scanned, and the lanes were plotted by ImageJ. The band intensities of holo-AcpP and reactive substrate 10:1(3-cis)-AcpP were integrated. Values reported are fraction of total band intensity corresponding to holo-AcpP. Reactions were performed in triplicate.

The FabI assay mixture containing 30 mM potassium phosphate (pH 7.4), 150 mM NaCl, 100 μM 10:1(2-trans)-AcpP, 500 μM NADH, and 0.012 μM FabI was set up for 50 μl per reaction. Each reaction was incubated at specific temperatures for 5 min to equilibrate before initiation by addition of NADH. 8 μl of each reaction mixture were quenched with 8 μl of 6x urea loading dye with 24 μl of Milli-Q Water at three different time points. Product formation was monitored on 15% urea-PAGE gels. Each gel was scanned and plotted by ImageJ. The band intensities of saturated decanoyl AcpP product and reactive substrate bands corresponding to 10:1(2-trans)-AcpP were integrated. Values reported are fraction of total band intensity corresponding to decanoyl-AcpP. Reactions were performed in triplicate.

### Mathematical modelling

Steady-state and dynamic simulations were performed using mathematical models constructed and evaluated using Python. Full details are provided in Supplemental Note 1 and Supplemental Note 2.

### Reporting summary

Further information on research design is available in the Nature Portfolio Reporting Summary linked to this article.

## Supplementary information

Supplementary Information

Reporting Summary

## Source data

Source Data

Transparent Peer Review file

## Data Availability

Source data are provided with this paper as a Source Data file. Raw LCMS data are available via Figshare (10.6084/m9.figshare.27144342.v1). Raw data from acyl-ACP and protein quantification by LCMS are also available from ProteomeXchange repository (project accession PXD051802). Metabolomics data have been deposited to the EMBL-EBI MetaboLights database^47^ with the identifier MTBLS11349. Source data are provided with this paper.

## Code availability

Python scripts used for mathematical models are available for download from zenodo.org/records/13908627 (DOI: 10.5281/zenodo.13908627).
