# Supplementary material for: A temperature-sensitive metabolic valve and a transcriptional feedback loop drive rapid homeoviscous adaptation in Escherichia coli
Source: Nat Commun. 2024 Oct 30;15:9386. doi: 10.1038/s41467-024-53677-5 (PMC11525553; doi:10.1038/s41467-024-53677-5)
Supplement: Supplementary file 1 — Supplementary Information [file 41467_2024_53677_MOESM1_ESM.pdf]

Supplementary Information

A temperature-sensitive metabolic valve and a transcriptional feedback loop drive rapid homeoviscous adaptation in *Escherichia coli*

Loles Hoogerland[1*], Stefan van den Berg[1*], Yixing Suo[2], Yuta W. Moriuchi[2], Adja Zoumaro-Djayoon[1], Esther Geurken[1], Flora Yang[1], Frank Bruggeman[3], Michael D. Burkart[2], Gregory Bokinsky[1]

[1] Department of Bionanoscience, Kavli Institute of Nanoscience, Delft University of Technology, Delft, The Netherlands.

[2] Department of Chemistry and Biochemistry, University of California, San Diego, CA, USA.

[3] Systems Biology Lab, AIMMS/ALIFE, Vrije Universiteit Amsterdam, Amsterdam, The Netherlands.

Supplemental Fig. 1

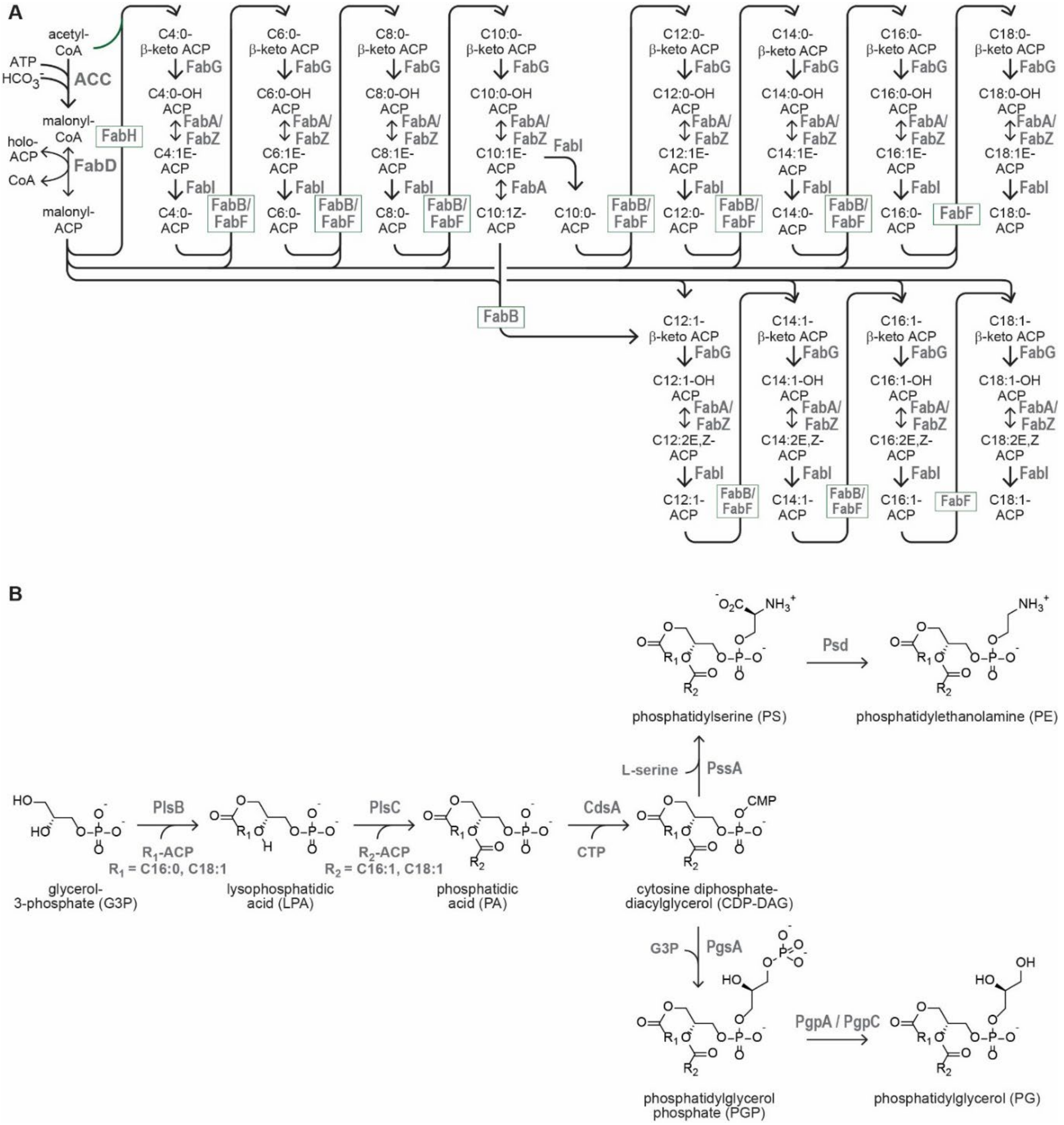

Supplemental Fig. 1. The complete *E. coli* fatty acid **(A)** and phospholipid synthesis pathways **(B)**, indicating enzymes catalysing each reaction.

Supplemental Fig. 2

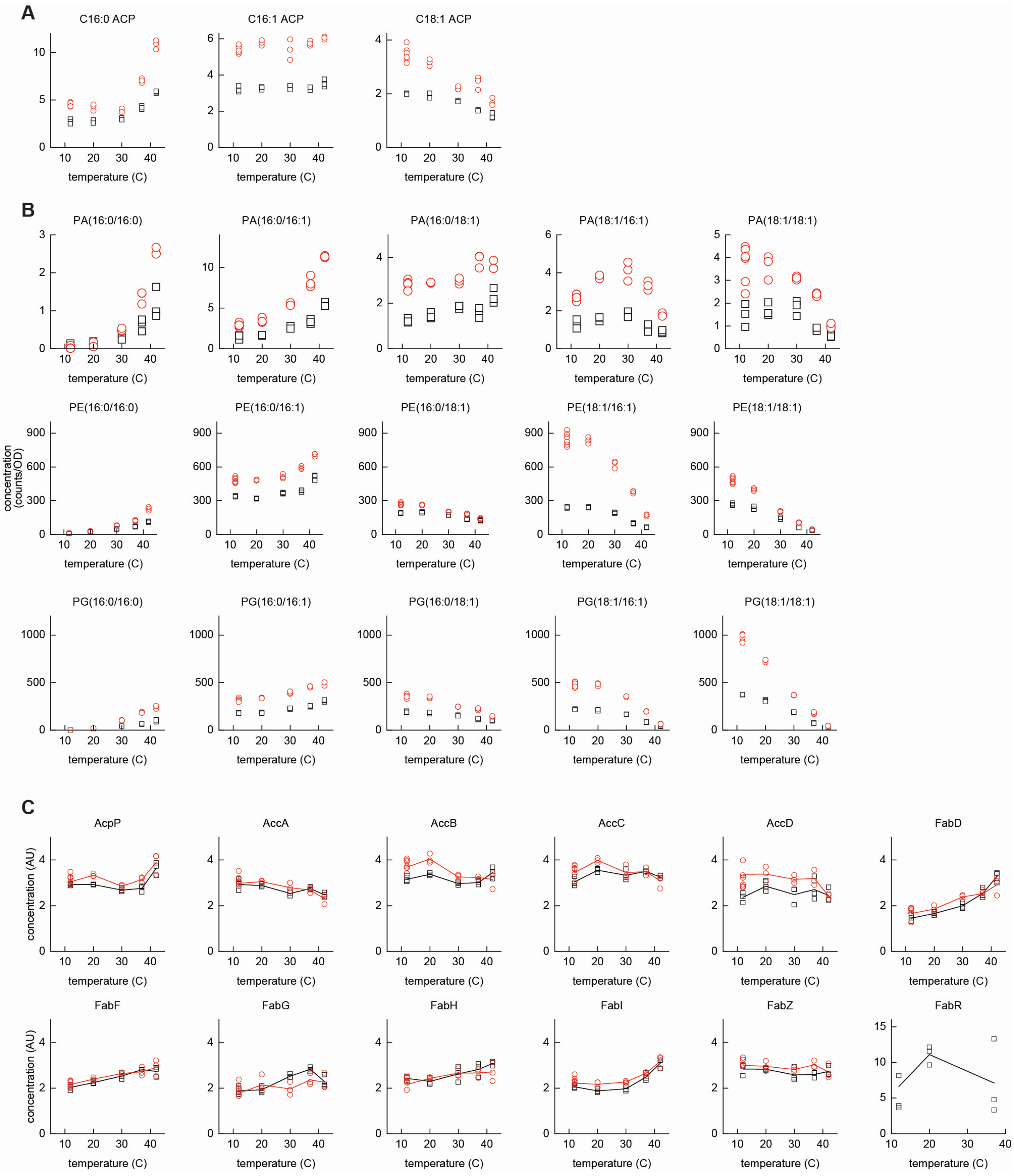

Supplemental Fig. 2. Abundance of acyl-ACP phospholipid precursors **(A)**, phospholipid intermediates and membrane phospholipids **(B),** and enzymes of the fatty acid synthesis pathway **(C)** across 5 temperatures. Data from 3 measurements from two independent cultures (n = 2) at each temperature (black and red symbols). FabR measurements from one culture (n = 1) at each temperature. Lines in **C** depict replicate averages. Source data are available as a Source Data File.

Supplemental Fig. 3

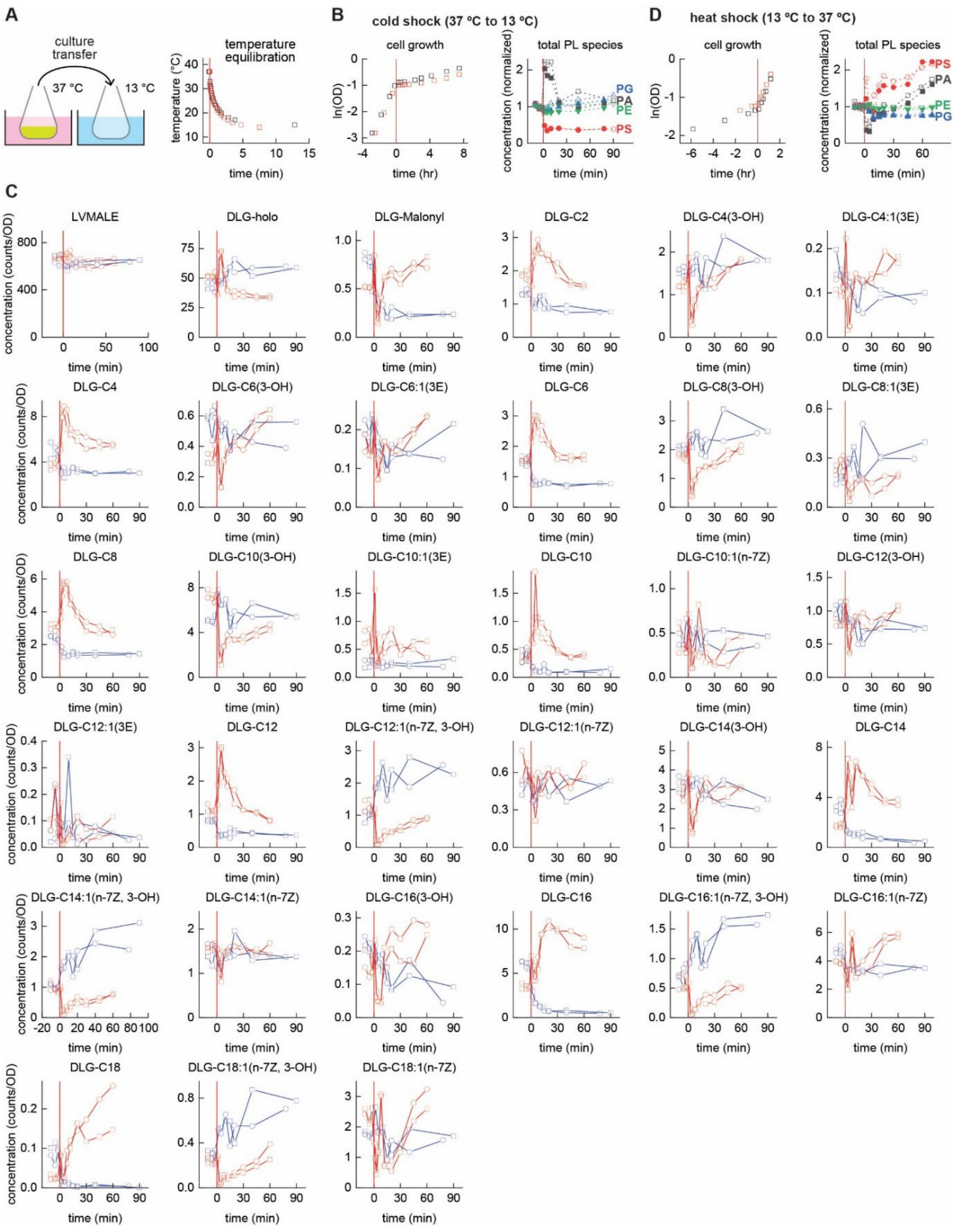

Supplemental Fig. 3. A. Exponential-phase cultures are transferred to empty flasks pre-incubated in a temperature-controlled bath, allowing rapid temperature equilibration. **B.** Effects of cold shock on growth and phospholipid abundance. **C.** Acyl-ACP concentrations following cold shocks (blue lines/symbols) and heat shocks (red lines/symbols). Two

independent cultures (n = 2) are shown for each temperature shock (one measurement per time point). **D.** Effects of heat shock on growth and phospholipid abundance. Source data are available as a Source Data File.

Supplemental Fig. 4

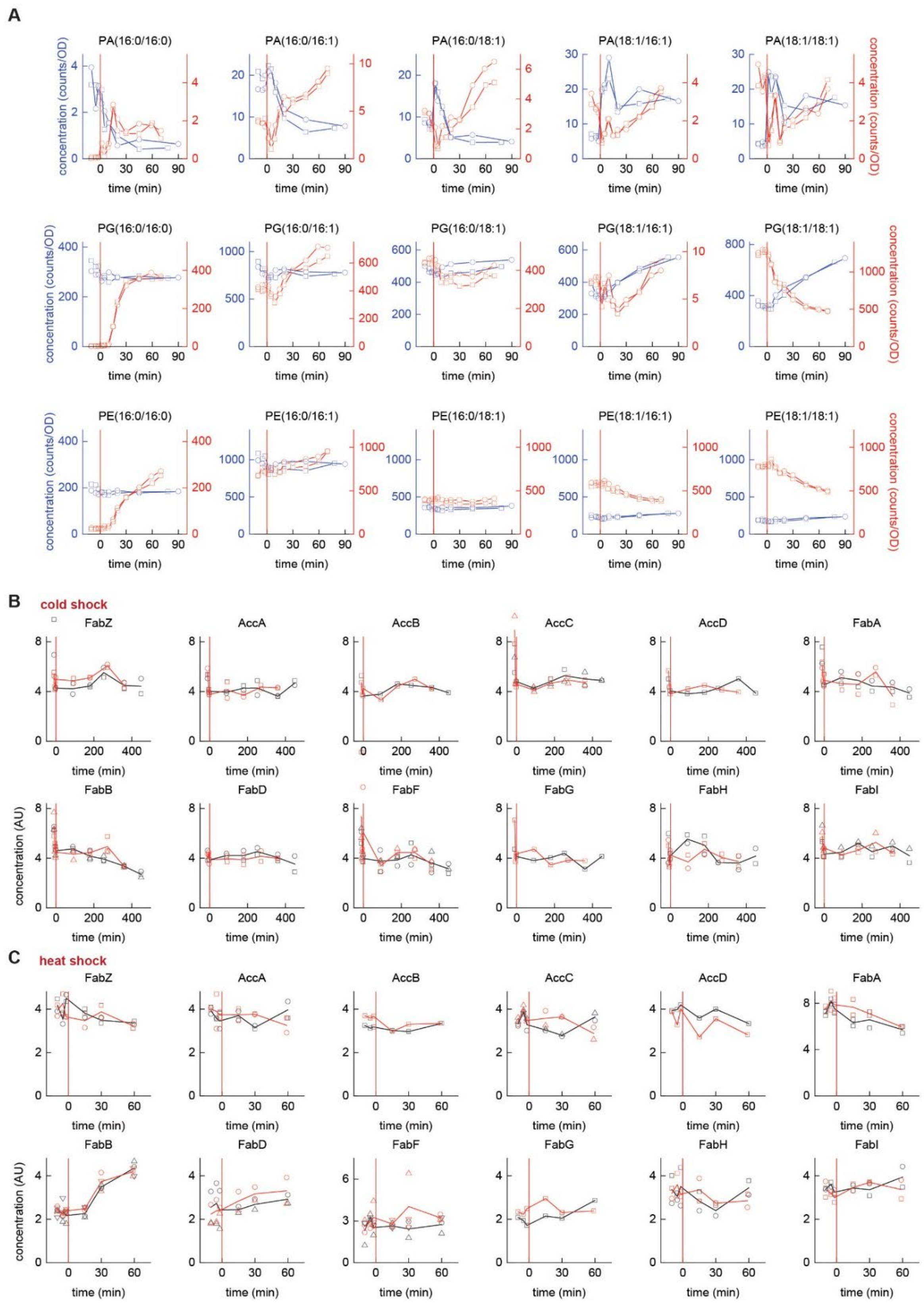

Supplemental Fig. 4. A. Concentrations of phospholipid synthesis intermediates and membrane phospholipids following cold shocks (blue lines/symbols) and heat shocks (red

lines/symbols). Data from 2 independent cultures (n = 2) are shown for each temperature shock. **B&C.** Concentrations of fatty acid synthesis pathway enzymes following cold shocks **(B)** and heat shocks **(C).** Data for 2 independent cultures (n = 2) are shown for each temperature shock. Scatter points depict concentrations of individual peptides relative to an internal standard, while lines indicates averages of relative peptide abundances for each replicate. Source data are available as a Source Data File.

Supplemental Fig. 5

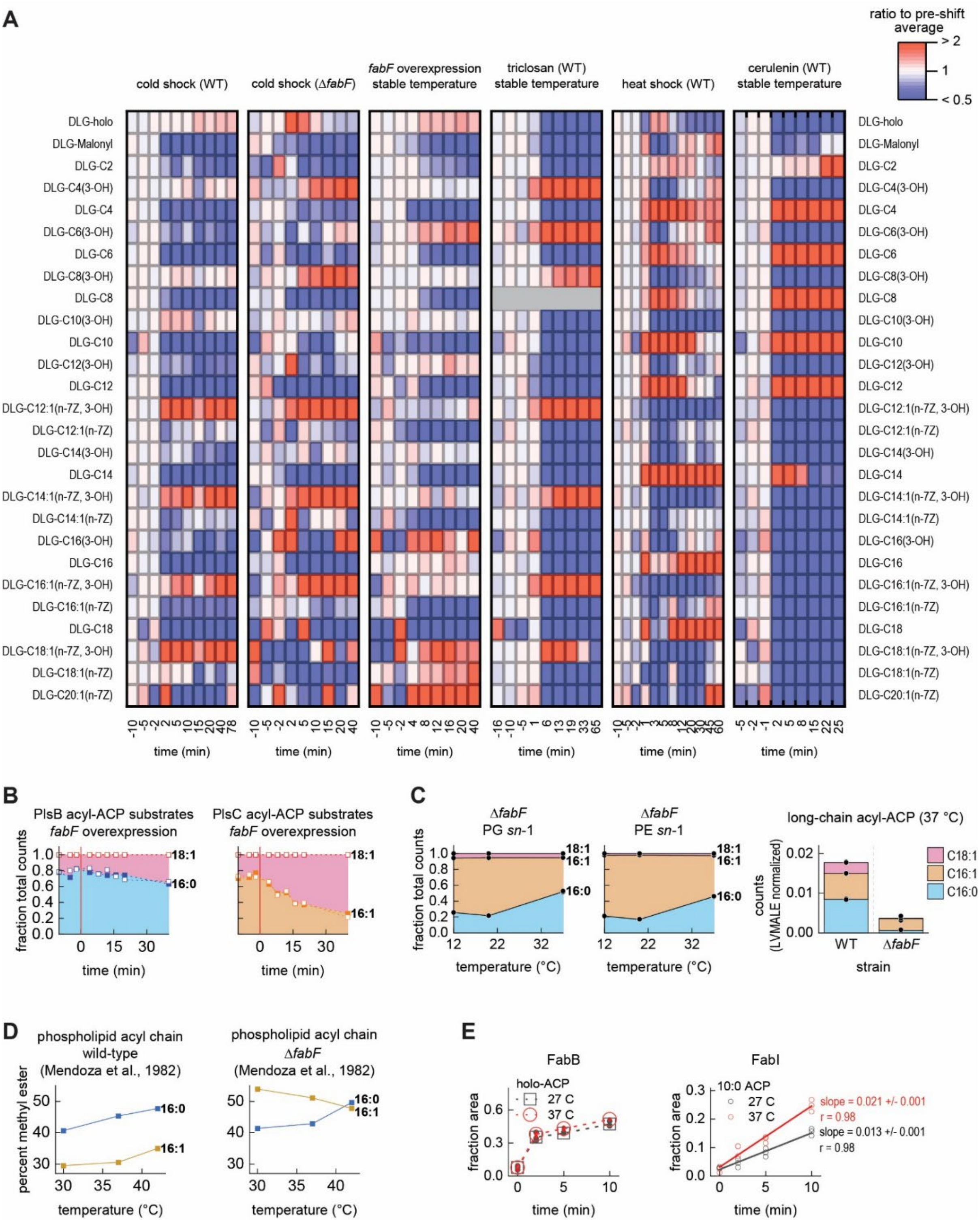

Supplemental Fig. 5. A. Responses of acyl-ACP pools to cold shock (wild-type and Δ*fabF*), heat shock, *fabF* overexpression, and triclosan treatment. Each pool is normalized to the average value of the first three time points (pre-treatment). To facilitate comparison, one replicate is shown for each condition. **B.** Composition of PlsB and PlsC substrate pools in wild-type *E. coli* overexpressing *fabF* from a plasmid (induced at 0 min). Kinetic series from

two biological replicates are shown (n = 2). **C.** Phospholipid composition of *E. coli* Δ*fabF* at three temperatures, indicating unusual incorporation of 16:1 acyl chain at *sn*-1 by PlsB. Abundance of long-chain acyl-ACP at 37 °C in wild-type and *E. coli* Δ*fabF.* Data shown are triplicate measurements (n = 3) of one culture at each condition. **D.** Phospholipid acyl chain composition of *E. coli* wild-type and Δ*fabF* reported in Table II of reference[1]. C14:0 and C18:1 values from the table not shown for clarity. **E.** *In vitro* activity of FabI and FabB analysed with C10:1(2*E*) and C10:1(3*Z*) ACP substrates at two temperatures. Depicted are product abundance over time. FabB: large open symbols represent averages of three experimental repeats (n = 3; small filled symbols). FabI: symbols represent three experimental repeats with linear fits. Source data are available as a Source Data File.

Supplemental Fig. 6

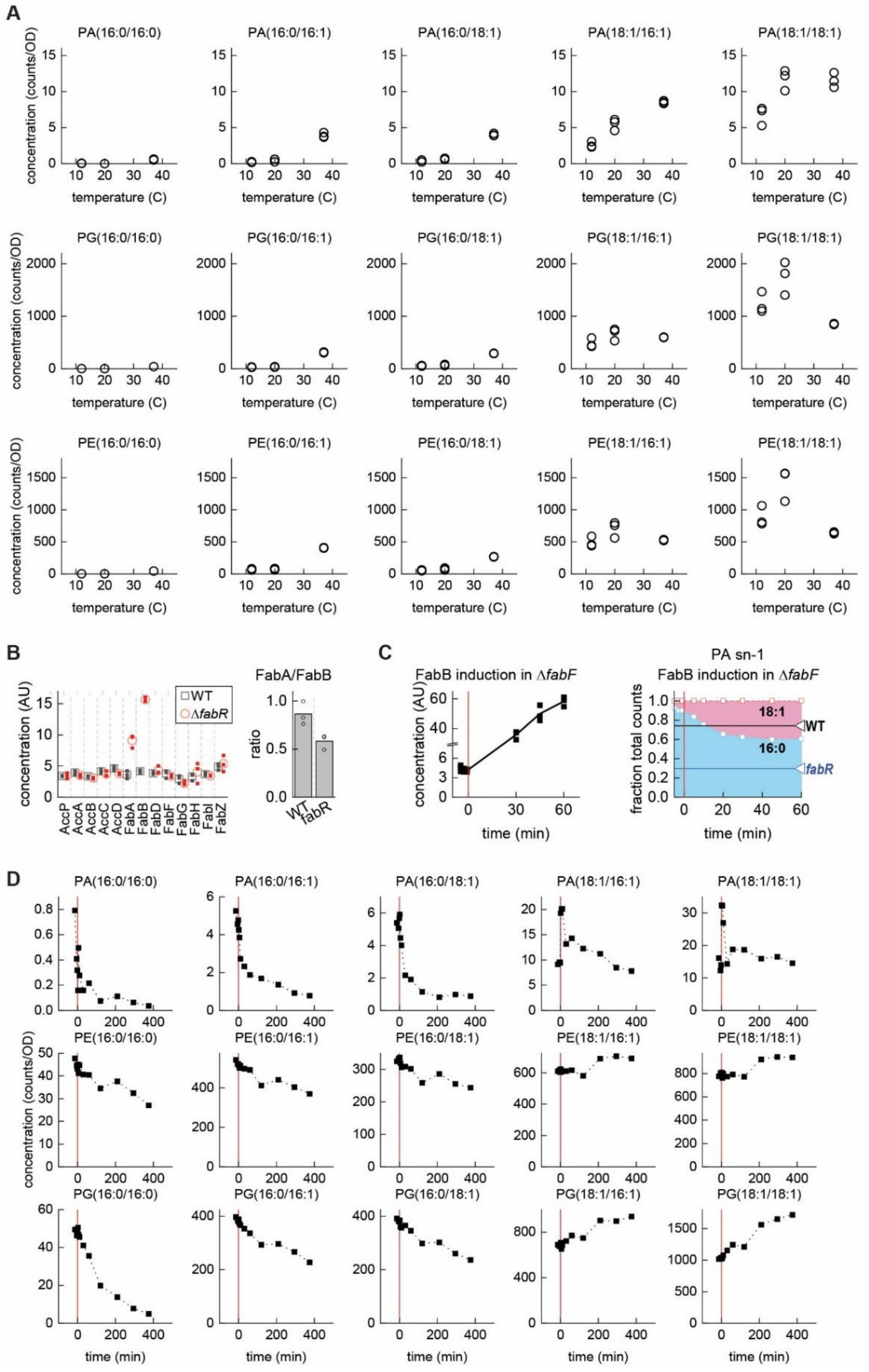

Supplemental Fig. 6. A. Steady-state phospholipid composition in *E. coli* Δ*fabR*. **B.** Concentration of fatty acid synthesis pathway enzymes and FabA/FabB ratio in *E. coli* wild-

type and Δ*fabR* at 37 °C*.* Open symbols represent averages of technical triplicates (small closed symbols). **C.** Concentration of FabB (left) and PA composition (right) in *E. coli* Δ*fabF* overexpressing *fabB* from a plasmid, induced at 0 min. Expression of FabB at approximately 15-fold above wild-type levels cannot reach membrane composition measured in *E. coli* Δ*fabR,* indicating that FabF is required to elongate C16:1 ACP and increase C18:1 phospholipids in *E. coli* Δ*fabR.* This demonstrates that the high abundance of unsaturated phospholipids observed in *E. coli* Δ*fabR* is due to increased FabB expression diverting more flux into the unsaturated pathway, not due to FabB elongating C16:1 ACP directly. **D.** Responses of individual phospholipid pools to cold shock in *E. coli* Δ*fabR*. All steady-state values reported are averages of technical replicates (n = 3) obtained from a single culture, as are FabB kinetic series displayed in **C**. Phospholipid kinetic series in **C** and **D** represent single timepoints from a single culture (n = 1). Source data are available as a Source Data File.

Supplemental Note 1

Simulating biochemical mechanisms that generate temperature-dependent flux redistribution. Experiments using *in vitro*-reconstituted fatty acids pathways from purified enzymes coupled with metabolic modelling indicate that the ratio of fluxes entering the saturated and unsaturated pathways are dependent not only upon FabB and FabI, but also upon activities of FabA and FabZ[2]. Inspired by these findings, we used simple metabolic models to identify mechanisms capable of redirecting flux towards saturated or unsaturated fatty acids in a temperature-dependent manner (Supplemental Fig. 7). The model describes a metabolic branchpoint with all four enzymes at the saturated/unsaturated branchpoint: acyl-ACP dehydratase FabZ, which reversibly dehydrates 3-hydroxydecanoyl ACP (C10:0-OH ACP, or DOH in this model) to C10:1(2*E*) ACP (DT); 2) a bifunctional dehydratase/isomerase FabA, which reversibly converts DOH to C10:1(3*Z*) ACP (DC); and FabB and FabI, which catalyze the committed step into the unsaturated and saturated branches, respectively. Phospholipids are synthesized as either unsaturated phospholipid (PU), by consuming two unsaturated fatty acids, or as saturated phospholipid (PS), by consuming one unsaturated and one saturated fatty acid. Phospholipids are subject to a slow consumption rate (μ), which reflects dilution by growth.

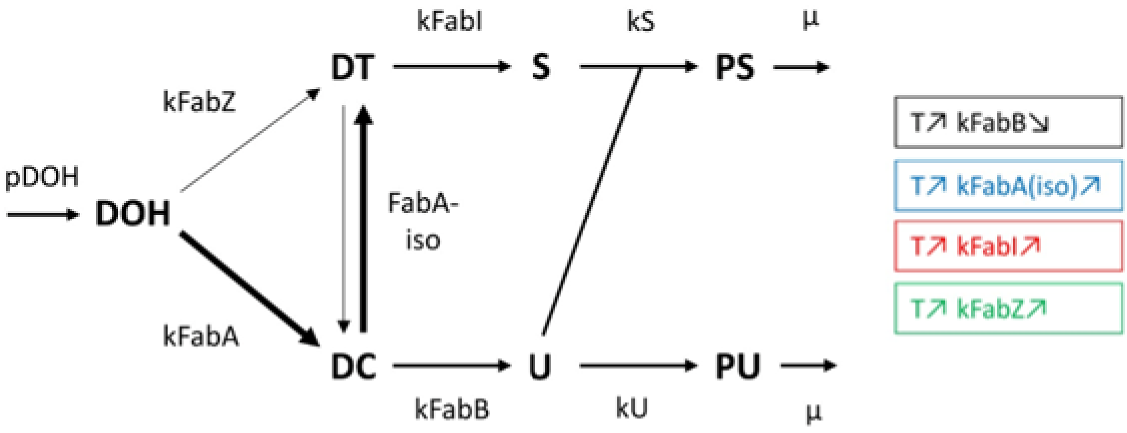

Supplemental Fig. 7. Overview of the model reactions, influence of temperature on specific rate constants, and parameter names.

The base model consists of the following differential equations:

$$\frac{dDOH(t)}{dt} = pDOH - k_{FabZ} \cdot c_{DOH}(t) - k_{FabA} \cdot c_{DOH}(t) \quad (1)$$

$$\frac{dDT(t)}{dt} = k_{FabZ} \cdot c_{DOH}(t) - k_{FabI} \cdot c_{DT}(t) - k_{FabAiso} \cdot c_{DT}(t) + k_{FabAiso} \cdot c_{DC}(t) \quad (2)$$

$$\frac{dDC(t)}{dt} = k_{FabA} \cdot c_{DOH}(t) - k_{FabB} \cdot c_{DC}(t) - k_{FabAiso} \cdot c_{DC}(t) + k_{FabAiso} \cdot c_{DT}(t) \quad (3)$$

$$\frac{dS(t)}{dt} = k_{FabI} \cdot c_{DT}(t) - k_S \cdot c_S(t) \cdot c_U(t) \quad (4)$$

$$\frac{dU(t)}{dt} = k_{FabB} \cdot c_{DC}(t) - (2 \cdot k_U \cdot c_U(t) \cdot c_U(t)) - (k_S \cdot c_S(t) \cdot c_U(t)) \quad (5)$$

$$\frac{dPS(t)}{dt} = k_S \cdot c_S(t) \cdot c_U(t) - \mu \cdot c_{PS}(t) \quad (6)$$

$$\frac{dPU(t)}{dt} = k_U \cdot c_U(t) \cdot c_U(t) - \mu \cdot c_{PU}(t) \quad (7)$$

Parameters are listed in Supplemental Table 1. Four different temperature dependent mechanisms were examined by updating the earlier set of equations with new ones for each model. The temperature range was set from hot (310 K) to cold (285 K), where the activity of k_{FabZ}, k_{FabA} and k_{FabI} decreased approximately 50% per 10 degrees K. In case of k_{FabB}, activity was decreased in the reverse manner (25% in hot versus cold temperature). The functions for temperature dependence are as follows:

$$k_{FabZ} = 0.1 \cdot \left(1 + \frac{8.22 \cdot (T-285)}{25}\right) \quad \text{(Model A)}$$

$$k_{FabA} = 3 \cdot \left(1 + \frac{8.22 \cdot (T-285)}{25}\right);\ k_{FabAiso} = 3 \cdot \left(1 + \frac{8.22 \cdot (T-285)}{25}\right) \quad \text{(Model B)}$$

$$k_{FabI} = 1 \cdot \left(1 + \frac{8.22 \cdot (T-285)}{25}\right) \quad \text{(Model C)}$$

$$k_{FabB} = 3 \cdot \left(1 - 0.25 \cdot \frac{(T-285)}{25}\right) \quad \text{(Model D)}$$

Activities resulting from these modelled activities are depicted in Supplemental Fig. 8.

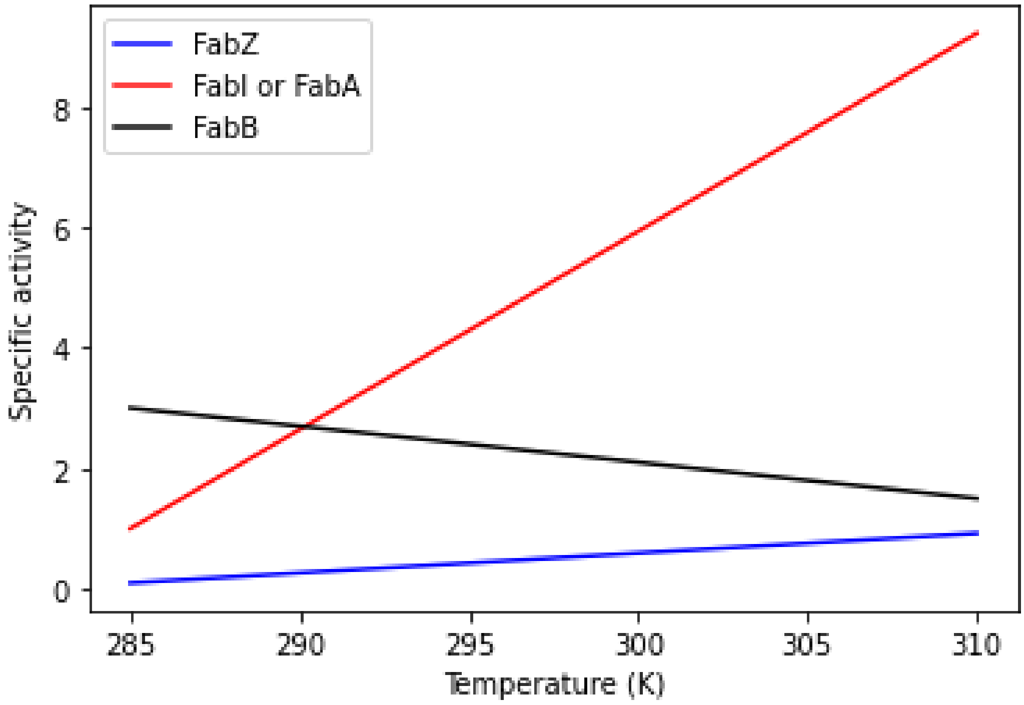

Supplemental Fig. 8. Changes in specific activity with temperature for Models A-D.

Supplemental Table 1. Parameter definitions and default values.

**Parameter**	**Value**	**Description**
pDOH	1	Production rate of DOH
k_{FabZ}	0.1	FabZ specific activity
k_{FabA}	1	FabA specific activity
$k_{FabAiso}$	1	FabA specific activity (isomerization)
k_{FabI}	1	FabI specific activity
k_{FabB}	3	FabB specific activity
k_S	1	Production rate of saturated phospholipids

k_U	1	Production rate of unsaturated phospholipids
μ	0.001	Growth rate
T	Variable	Temperature

Introducing temperature dependence into the specific activity of individual enzymes while maintaining all others at a fixed value causes the fraction of saturated phospholipids to vary with temperature (Supplemental Fig. 9).

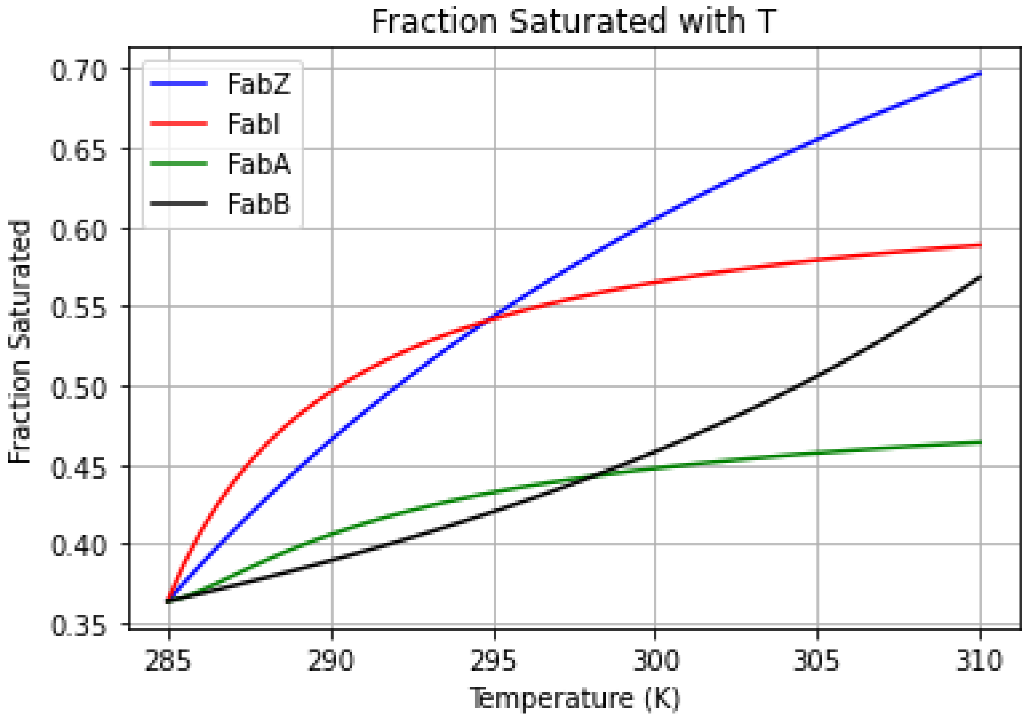

Supplemental Fig. 9. Saturated phospholipid fractions resulting from varying specific enzyme activities.

Supplemental Note 2

Mathematical model of temperature-sensitive metabolic branch point combined with transcriptional feedback loop. We modelled the temperature response of the biosynthesis of saturated fatty acids (produced by FabI) and unsaturated fatty acids (produced by FabB) and their corresponding saturated and unsaturated phospholipid products with a minimal model. In addition to a temperature dependence of the catalytic rate constants of both FabI and FabB, this model considers the transcriptional regulation of FabB by its transcription factor FabR, whose activity depends on the concentration of saturated and unsaturated fatty acids (Supplemental Fig. 10). We considered the dimerization of this transcription factor as well as the binding of its two regulatory fatty acids.

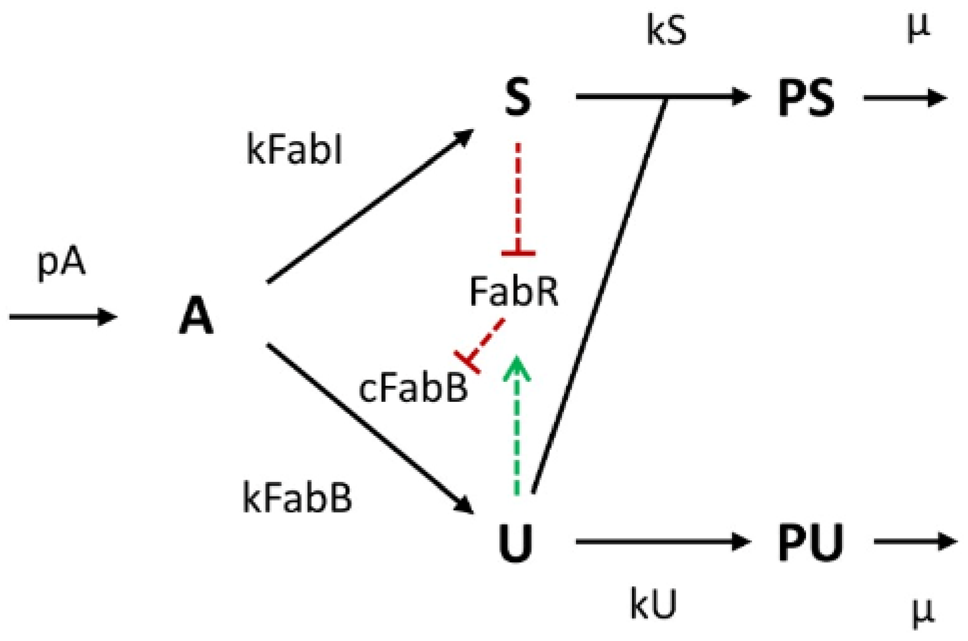

Supplemental Fig. 10. Overview of the system captured by the kinetic model.

A precursor (A) is produced at a constant rate (p_A). This precursor can be converted into either a saturated fatty acid (S) by FabI or an unsaturated fatty acid (U) by FabB. Subsequently, these fatty acids are converted to phospholipids. A saturated phospholipid (PS) consists of one S and one U, whereas an unsaturated phospholipid (PU) consists of two Us. FabR regulates the concentration of FabB.

The model consists of the following differential equations:

$$\frac{dS(t)}{dt} = k_{FabI} \cdot c_{FabI}(t) \cdot c_A(t) - k_S \cdot c_S(t) \cdot c_U(t) \quad (8)$$

$$\frac{dU(t)}{dt} = k_{FabB} \cdot c_{FabB}(t) \cdot c_A(t) - (2 \cdot k_U \cdot c_U(t)^2) - (k_S \cdot c_S(t) \cdot c_U(t)) \quad (9)$$

$$\frac{dA(t)}{dt} = p_A - k_{FabI}(t) \cdot c_{FabI}(t) \cdot c_A(t) - k_{FabB}(t) \cdot c_{FabB}(t) \cdot c_A(t) \quad (10)$$

$$\frac{dPS(t)}{dt} = k_S \cdot c_S(t) \cdot c_U(t) - \mu \cdot c_{PS}(t) \quad (11)$$

$$\frac{dPU(t)}{dt} = k_U \cdot c_U(t)^2 - \mu \cdot c_{PU}(t) \quad (12)$$

$$\frac{dFabB(t)}{dt} = \frac{k_t}{1+\left(\frac{FabR_2U_2(t)}{K_r}\right)^2} - \mu \cdot c_{FabB}(t) \quad (13)$$

$$FabR_2U_2(t) = \frac{\left(2FabR_T+K_{FabR}{}^2-\sqrt{K_{FabR}{}^2(4FabR_T+K_{FabR}{}^2)}\right)K_S{}^2u(t)^2}{2(K_SK_U+K_Us(t)+K_Su(t))^2} \quad (14)$$

The temperature dependency of the catalytic rate constants $k_{FabI}(T)$ and $k_{FabB}(T)$ as function of temperature are shown in Figure 4C of the main text. The dependency is based on a skewed normal distribution using $\alpha_{FabI} = -30, loc_{FabI} = 320, scale_{FabI} = 15, \alpha_{FabB} = -30, loc_{FabB} = 320, scale_{FabI} = 40$. The shape of these functions resembles experimentally-determined temperature dependencies of enzymatic activities reported in the literature[3]. The decrease of about 50% in FabI catalytic activity from 310K to 300K resembles our *in vitro* results obtained for the temperature dependence of FabI activity. Parameters used in the simulation are given in Supplemental Table 2.

Supplemental Table 2. Parameters used in the simulation.

**Parameter**	**Value**	**Description**
k_S	0.1	Rate of saturated phospholipid production
k_U	0.1	Rate of unsaturated phospholipid production
μ	0.001	Growth rate
K_{FABR}	0.1	Dimerization equilibrium constant of FabR

$FabR_T$	1	Total concentration of FabR
K_S	0.5	Disssociation constant of FabR dimer for S
K_U	0.1	Disssociation constant of FabR dimer for U
K_r	0.12	Dissociation constant of FabB promotor
k_t	0.007	Transcription rate of FabB
p_A	2	Production rate of precursor A
c_{FabI}	0.5	Constitutive expression level of FabI
T_{hot}	310	Hot shock temperature in Kelvin
T_{cold}	285	Cold shock temperature in Kelvin

The model is written as a Python code. A system of ordinary differential equations (ODE's) is defined and solved using the odeint solver from the scipy.integrate package. An initial run was executed to find the steady state concentration values at 285 K as input for subsequent runs. The model describes the bound states of the transcription factor FabR with unsaturated and saturated fatty acid as function of the concentrations of those fatty acids, described both the dimerization of the transcription factor and the fatty acid binding events.

The dimerization is described by

$$FabR_T = FabR + 2\ FabR_{2T} \qquad (15)$$

$$FabR_{2T} = \frac{FabR^2}{K_{FABR}} \qquad (16)$$

which leads to the following equilibrium concentration of the dimer,

$$FabR_{2T} = \frac{1}{8}\left(4\ FabR_T + K_{FABR} - \sqrt{K_{FABR}(8\ FabR_T + K_{FABR})}\right) \qquad (17)$$

This total dimer concentration occurs in different bound states,

$$FabR_{2T} = FabR_2 + 2FabR_2S + 2FabR_2U + 2FabR_2US + FabR_2S_2 + FabR_2U_2 \quad (18)$$

And the following associated binding equilibrium relations

$$FabR_2S = FabR_2\frac{s(t)}{K_S}, \qquad (19)$$

$$FabR_2S = FabR_2\frac{u(t)}{K_S}, \qquad (20)$$

$$FabR_2US = FabR_2\frac{u(t)s(t)}{K_SK_U} \qquad (21)$$

$$FabR_2U_2 = FabR_2\left(\frac{u(t)}{K_U}\right)^2, \qquad (22)$$

$$FabR_2S_2 = FabR_2\left(\frac{s(t)}{K_S}\right)^2 \qquad (23)$$

All these equations can be solved for the transcription regulating state of FabR.

$$FabR_2U_2 = FabR_{2T}\frac{u(t)^2{K_S}^2}{\left(K_SK_U+K_Us(t)+K_Su(t)\right)^2} \qquad (24)$$

As the model is highly simplified and lumps together multiple processes and physiological parameters, we simulated the model with normalized time and concentration units. The model captures the essence of the regulatory system.

Supplemental Note 3

Preparation of acyl-ACP substrates for *in vitro* experiments.

Part I: Preparation of substrates

(2E,4E)-Deca-2,4-dienal, trans-2-decenoic acid, and decanoic acid were commercially purchased from AmBeed, TCI, and Fluka respectively. Solvents were purchased from Fisher Scientific. ^{1}H-NMR and ^{13}C-NMR was collected on JEOL 400. NMR files were processed with MestRaNova Version 14.2.2. The HR-MS was collected on ThermoFisher LTQ Orbitrap XL at the UCSD Molecular Mass Spectrometry Facility.

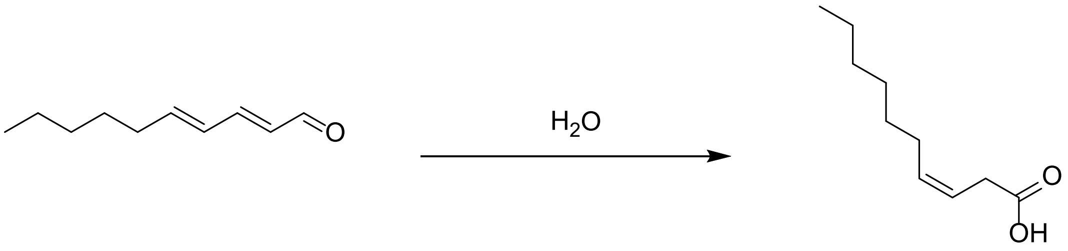

S1

Cis-3-decenoic acid (S1): Cis-3-decenoic acid was prepared in a similar manner to a previously published procedure[4]. In a 25 mL microwave reactor tube 2.8 g of (2E,4E)-deca-2,4-dienal was added to 8 mL of H_2O. The reaction was heated to 230 °C for 30 minutes, and then allowed to cool. Once cooled to 55 °C, the reaction was extracted three times with 10 mL diethyl ether and the combined organic layers were dried over Na_2SO_4 and concentrated by rotatory evaporation. The crude was purified by silica gel chromatography using a gradient of 100 % hexanes to 100 % diethyl ether. A portion of the compound was further purified by preparatory TLC resulting in 24.7 mg of cis-3-decenoic acid. ^{1}H NMR (400 MHz, CHLOROFORM-*D*) δ 5.66 – 5.47 (m, 2H), 3.13 (dd, *J* = 7.0, 1.4 Hz, 2H), 2.03 (q, *J* = 7.1 Hz, 2H), 1.41 – 1.31 (m, 2H), 1.35 – 1.25 (m, 2H), 1.29 – 1.19 (m, 6H), 0.90 – 0.83 (m, 3H). ^{13}C NMR (100 MHz, CHLOROFORM-*D*) δ 178.32, 134.32, 119.95, 32.68, 31.79, 29.31, 28.99, 27.50, 22.71, 14.17. HR-MS (ESI -) Calculated: 169.1234; Found: 169.1231; Δ -1.8 ppm $[C_{10}H_{17}O_2]^-$

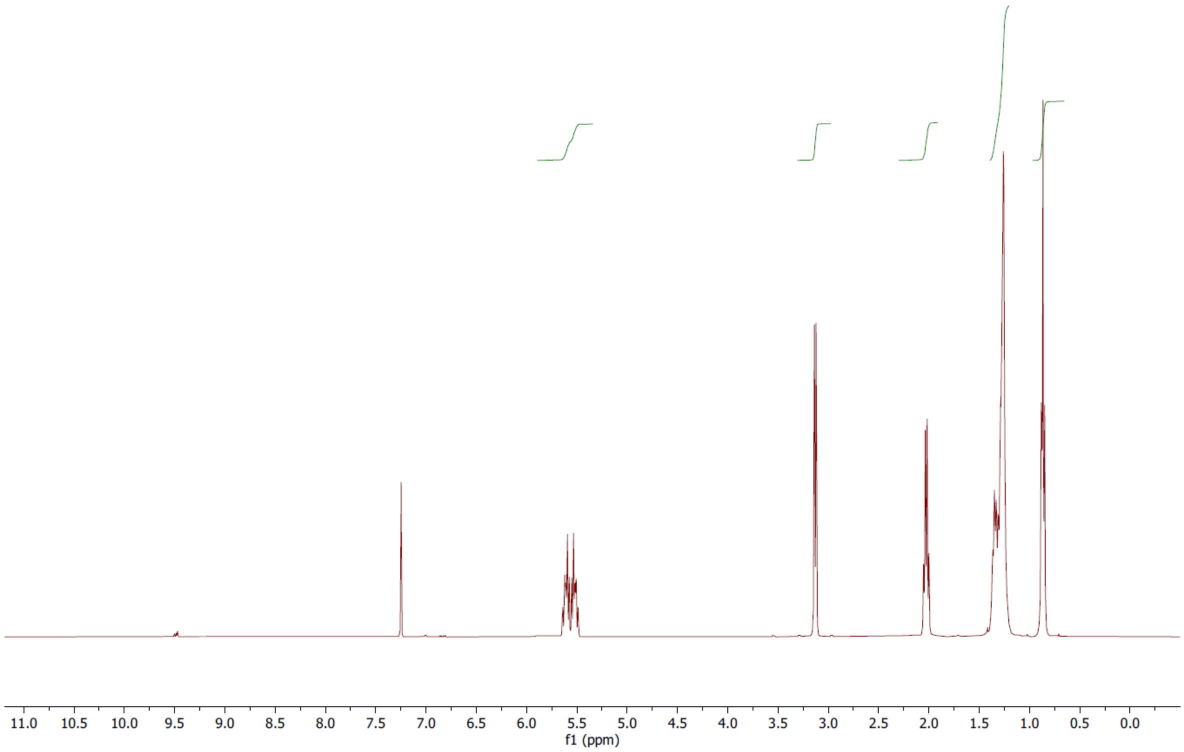

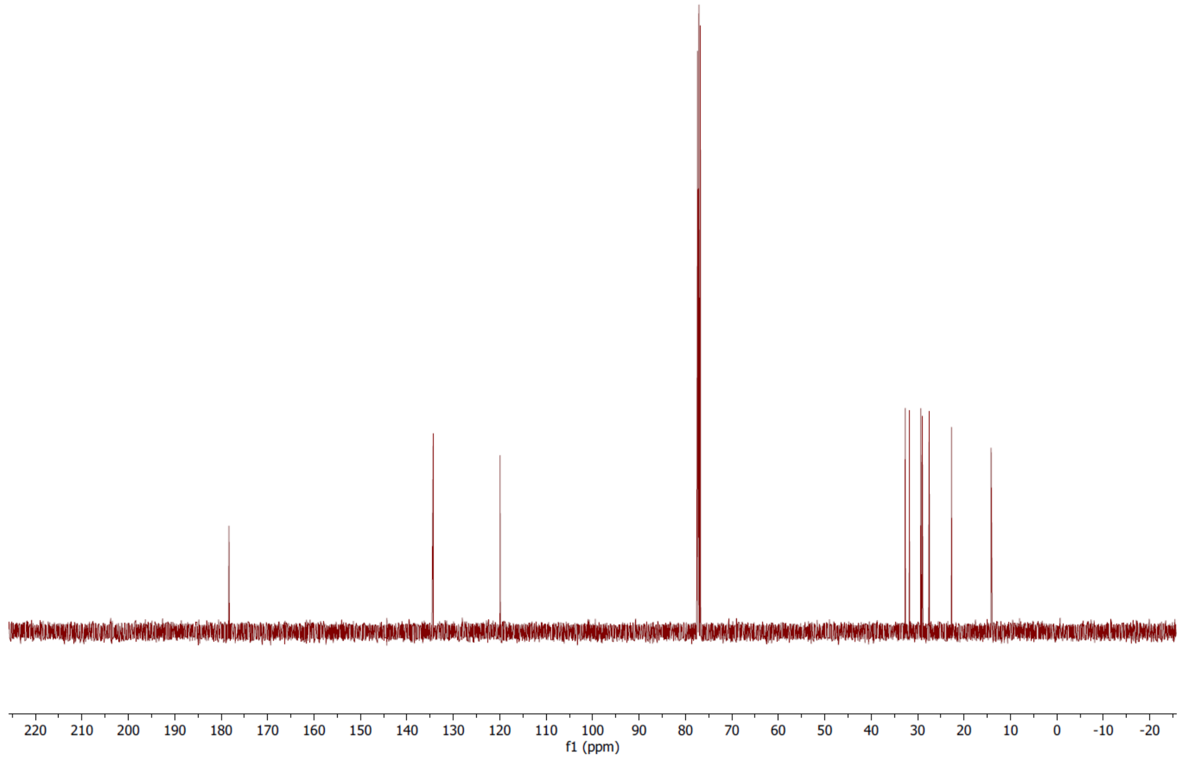

Part II: Substrates loading

The substrate loading reactions were performed according to a previously published procedure.[5] Briefly, *holo*-AcpP was converted to the corresponding *acyl*-AcpP with a reaction mixture of 100 μM *holo*-AcpP, 1 μM *V. harveyi* acyl-ACP synthetase (AasS), 1 mM of the corresponding substrate, 2 mM ATP, 12.5 mM $MgCl_2$ at room temperature for 2 hours. The proteins were subsequently purified through gel filtration.

Supplemental Table 3

Supplemental Table 1. Primer sequences

Name	Sequence	Used for
GB220426-fabF.f	aaaGAATTC GGAGGACAAACgtgTCTAAGCG	fabF cloning
GB220426-fabF.r	ttactcgagTTAGGATCCttaGATCTTTTTAAAGATCAAAGAACCATTAGTGC	fabF cloning
GB220426-fabB.f	aaaAGATCT GCGACTTACAGAGGTATTGAatgAAAC	fabB cloning
GB220426-fabB.r	ttactcgagTTAGGATCCttaATCTTTCAGCTTGCGCATTACC	fabB cloning
GB220426-fabA.f	aaaAGATCT AAATAAGGCTTACAGAGAACatgGTAG	fabA cloning
GB220426-fabA.r	ttactcgagTTAGGATCCtcaGAAGGCAGACGTATCCTGG	fabA cloning
GB230102fabFKO.f1	CTAGAATCATTTTTTCCCTCCCTGGAGGACAAACgtg gtgtaggctggagctgcttc	fabF knockout
GB230102fabFKO.f2	GTCGTTCGACCGCCTGAGTTTTATCTTTTTGTCCCACTAGAATCATTTTTTCCCTCCC	fabF knockout
GB230102fabFKO.r1	GTGGAAAATGACAACttaGATCTTTTTAAAGATCAAatgggaattagccatggtcc	fabF knockout
GB230102fabFKO.r2	GGCCCGCAAGCGGACCTTTTATAAGGGTGGAAAATGACAACttaGATC	fabF knockout
GB230105-fabFKO.fseq	CAGGCGTAAGTGAACATCTCC	confirming fabF knockout
GB230105-fabFKO.rseq	GTTCACTACGGAACAAGTCGG	confirming fabF knockout
GB230102fabRKO.f1	CCGTTCATTCACAATACTGGAGCAATCCAGTatg gtgtaggctggagctgcttc	fabR knockout
GB230102fabRKO.f2	CAAAGAATTGCAGTAAATATGTTTTATTGCGTTACCGTTCATTCACAATACTGG	fabR knockout
GB230102fabRKO.r1	GCTTGTTTCAttaCTCGTCCTTCACATTTCCatgggaattagccatggtcc	fabR knockout
GB230102fabRKO.r2	CTAACGCCAGCAGCAGCGTACCTCTATCTTGATTTGCTTGTTTCAttaCTCGTCC	fabR knockout
GB230105-fabRKO.fseq	GCAGGTTTCCGCTGTACG	confirming fabR knockout
GB230105-fabRKO.rseq	AGTACCATTAATCGATAAGCCAGC	confirming fabR knockout

Supplementary References

1. De Mendoza, D., Klages Ulrich, A. & Cronan, J. E. Thermal regulation of membrane fluidity in Escherichia coli. Effects of overproduction of β-ketoacylacyl carrier protein synthase. *J. Biol. Chem.* **258**, 2098–2101 (1983).

2. Ruppe, S., Mains, K. & Fox, J. M. A kinetic rationale for functional redundancy in fatty acid biosynthesis. *Proc. Natl. Acad. Sci. U. S. A.* **117**, 23557–23564 (2020).

3. Peterson, M. E., Daniel, R. M., Danson, M. J. & Eisenthal, R. The dependence of enzyme activity on temperature: determination and validation of parameters. *Biochem. J.* **402**, 331–337 (2007).

4. Chen, X. *et al.* One-step and non-catalytic intramolecular redox reactions of conjugated all E-dienals to non-conjugated Z-enoic acids in subcritical water. *J. Supercrit. Fluids* **62**, 178–183 (2012).

5. Zhu, L., Zou, Q., Cao, X. & Cronan, J. E. Enterococcus faecalis encodes an atypical auxiliary acyl carrier protein required for efficient regulation of fatty acid synthesis by exogenous fatty acids. *MBio* **10**, (2019).
