# Supplementary material for: A temperature-sensitive metabolic valve and a transcriptional feedback loop drive rapid homeoviscous adaptation in Escherichia coli
Source: Nat Commun. 2024 Oct 30;15:9386. doi: 10.1038/s41467-024-53677-5 (PMC11525553; doi:10.1038/s41467-024-53677-5)
Supplement: Supplementary file 4 — Transparent Peer Review file [file 41467_2024_53677_MOESM4_ESM.zip › Source Data/FabB and FabI Assay Gels.pdf]

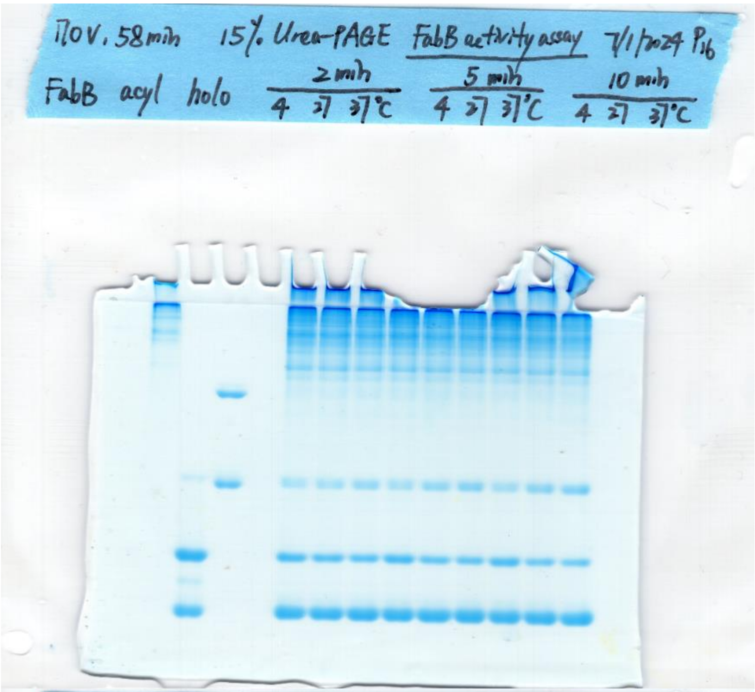

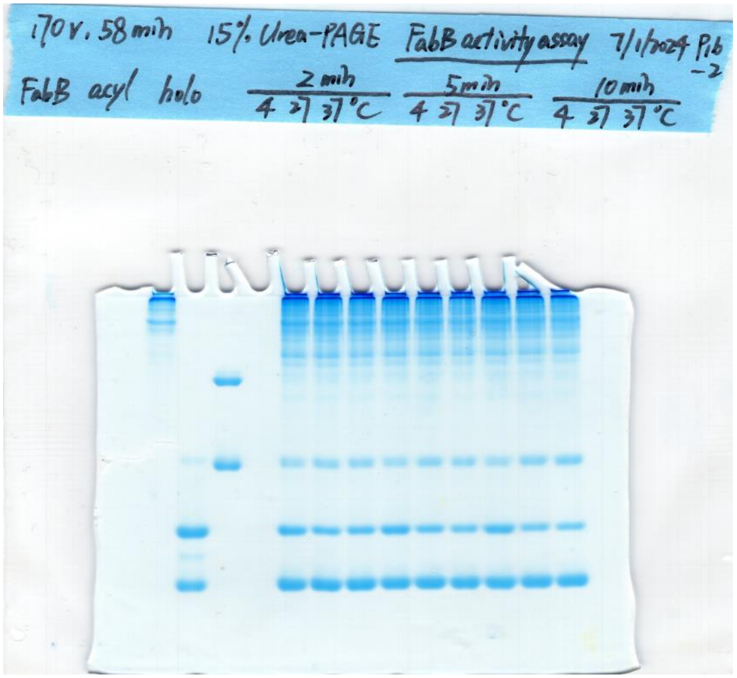

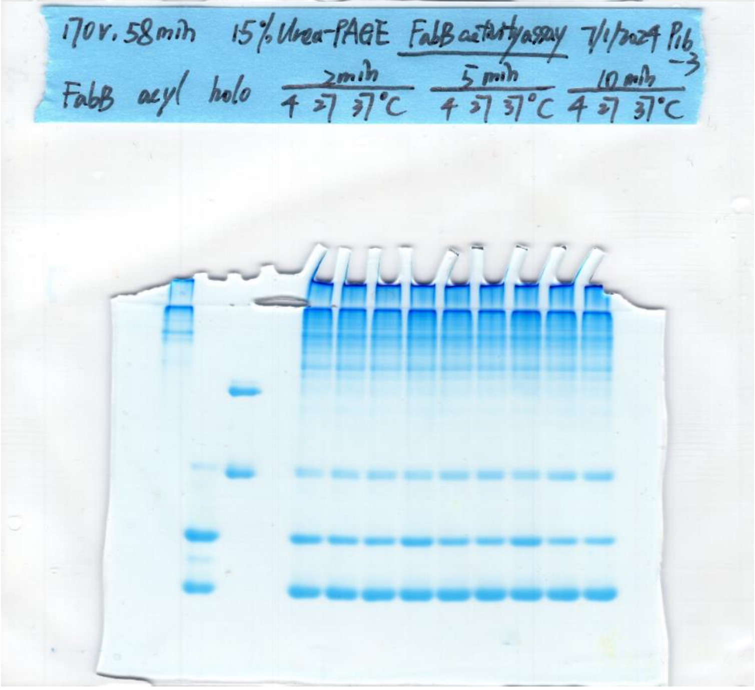

FabB Assay Gels

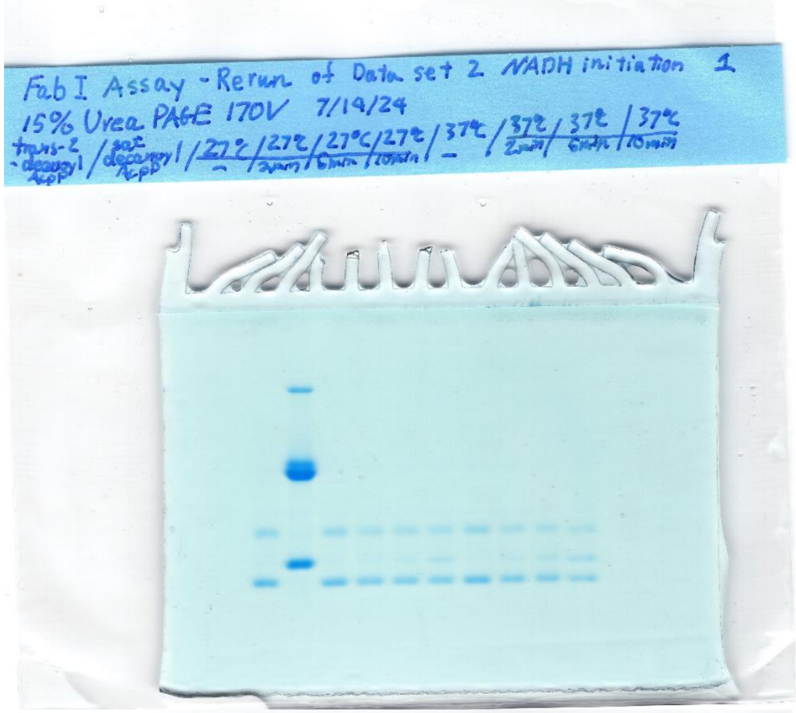

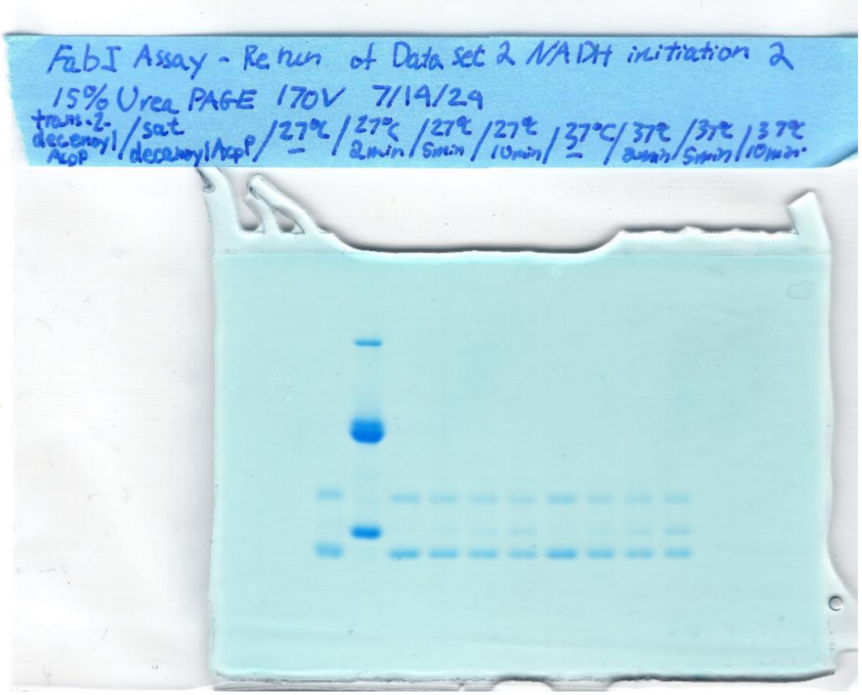

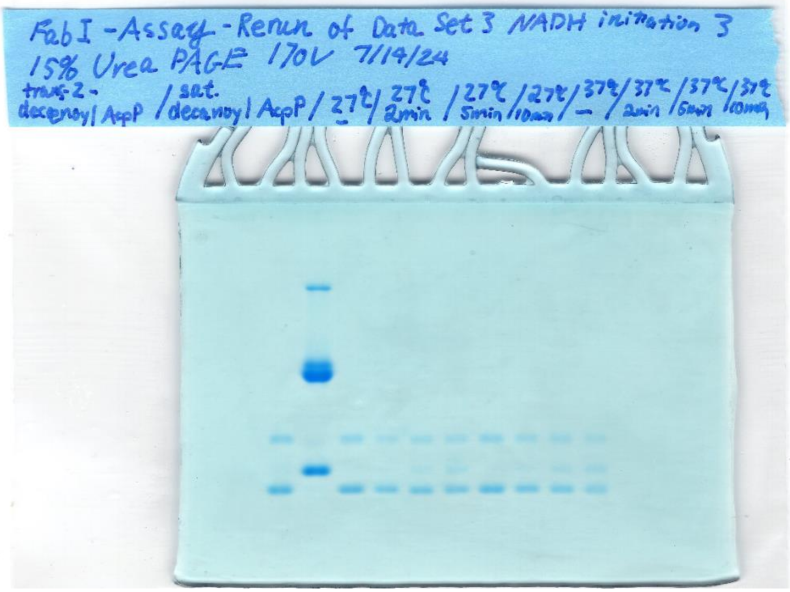

FabI Assay Gels
